# An Efficient Narrowband Near‐Infrared at 1040 nm Organic Photodetector Realized by Intermolecular Charge Transfer Mediated Coupling Based on a Squaraine Dye

**DOI:** 10.1002/adma.202100582

**Published:** 2021-05-31

**Authors:** Jin Hong Kim, Andreas Liess, Matthias Stolte, Ana‐Maria Krause, Vladimir Stepanenko, Chuwei Zhong, David Bialas, Frank Spano, Frank Würthner

**Affiliations:** ^1^ Universität Würzburg Center for Nanosystems Chemistry (CNC) and Bavarian Polymer Institute (BPI) Theodor‐Boveri‐Weg 97074 Würzburg Germany; ^2^ Universität Würzburg Institut für Organische Chemie Am Hubland 97074 Würzburg Germany; ^3^ Department of Chemistry Temple University 130 Beury Hall, 1901 N. 13th Street Philadelphia PA 19122 USA

**Keywords:** crystal engineering, J‐aggregates, near‐infrared sensitivity, organic photodiodes, squaraine dyes

## Abstract

A highly sensitive short‐wave infrared (SWIR, λ > 1000 nm) organic photodiode (OPD) is described based on a well‐organized nanocrystalline bulk‐heterojunction (BHJ) active layer composed of a dicyanovinyl‐functionalized squaraine dye (**SQ‐H**) donor material in combination with **PC_61_BM**. Through thermal annealing, dipolar **SQ‐H** chromophores self‐assemble in a nanoscale structure with intermolecular charge transfer mediated coupling, resulting in a redshifted and narrow absorption band at 1040 nm as well as enhanced charge carrier mobility. The optimized OPD exhibits an external quantum efficiency (EQE) of 12.3% and a full‐width at half‐maximum of only 85 nm (815 cm^−1^) at 1050 nm under 0 V, which is the first efficient SWIR OPD based on J‐type aggregates. Photoplethysmography application for heart‐rate monitoring is successfully demonstrated on flexible substrates without applying reverse bias, indicating the potential of OPDs based on short‐range coupled dye aggregates for low‐power operating wearable applications.

## Graphical abstract

Narrowband near‐infrared organic photodiodes are reported based on a dipolar squaraine dye. J‐type coupling in the solid state allows **SQ‐H** thin films to combine favorable NIR absorption at 1040 nm and charge carrier mobility. The bulk‐heterojunction with **PC_61_BM** yields an organic photodiode with external quantum efficiency of 12.3% at 1050 nm with a full‐width half‐maximum of 85 nm under short‐circuit condition.

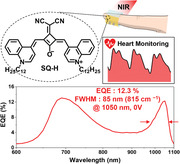

## 1 Introduction

Near‐infrared (NIR) photodetectors have attracted a growing attention due to their potential applications in emerging innovative technologies such as machine vision,^[^ ^1^ ^]^ optical communication,^[^ ^2^ ^]^ spectroscopy for chemical fingerprinting,^[^ ^3^ ^]^ and biomedical monitoring.^[^ ^4^ ^]^ These new applications require tailor‐made photodetector characteristics originating from optimized optoelectronic performance of new materials as well as a smart device architecture design.^[^ ^5^ ^]^ Particularly, sensitive short‐wave infrared (SWIR) organic photodiodes (OPDs) in the wavelength (λ) range from 1000 to 3000 nm, along with narrow spectral responses of <100 nm full‐width at half‐maximum (FWHM) are desirable for biomedical applications due to the deep penetration depth in biotissues and a reduced background signal from the environment.^[^ ^4b^ ^]^ Most commercially available photodetectors are based on single‐crystalline inorganic semiconductors including silicon (Si) and indium gallium arsenide (InGaAs), which generally have limited optical tunability and low absorption coefficients.^[^ ^5^, ^6^ ^]^ These drawbacks require thick active layers and additional optical filters for a sufficient photoresponse for specific wavelengths and thereby limit their applicability in flexible and stretchable devices due to the inherent rigid nature of thick inorganic semiconductor layers.

In this regard, organic dyes are emerging as alternative photoactive materials due to their tunable optical properties through molecular design and crystal engineering as well as inherently high absorption coefficient. Moreover, these organic semiconductors are suitable for inexpensive wearable applications because their thin active layers are often solution‐processable and accordingly flexible as well as light‐weight.^[^ ^5c^ ^]^ Indeed, numerous excellent panchromatic bulk‐heterojunction (BHJ) OPDs have been reported based on established organic semiconductors developed for high‐performing organic solar cells including nonfullerene acceptors.^[^ ^7^ ^]^ However, only a handful OPDs have been reported with absorption bands in the SWIR region, and most of them exhibit an external quantum efficiency (EQE) of less than 10%.^[^ ^8^ ^]^ In addition, all of them show unselective and broadband absorption with FWHM > 100 nm (Figure S1 and Table S1, Supporting Information). To obtain a narrow spectral response from broadband OPDs, several elegant device design strategies, such as charge collection narrowing^[^ ^9^ ^]^ and microcavity‐induced narrowing,^[^ ^3^, ^10^ ^]^ were proposed. However, it is still challenging to achieve an EQE over 10% in SWIR region, because these top‐down approaches require not only precisely controlled device structures, but also optically suitable BHJ materials for each strategy.

As a bottom‐up approach, utilizing supramolecular engineering to form J‐type aggregates is another promising strategy for NIR photodetectors because the exchange narrowing between the chromophores can result in strongly redshifted absorption spectra with narrow FWHM, respectively.^[^ ^11^ ^]^ Indeed, previously we successfully demonstrated ultranarrowband OPDs based on supramolecular structures of dipolar merocyanine dyes, one of which showed a narrow absorption band at 749 nm with FWHM of only 36 nm (630 cm^−1^).^[^ ^12^ ^]^ Likewise, several J‐type aggregates have been reported for NIR photodetectors, but all showed maximum absorption wavelengths of less than 1000 nm.^[^ ^13^ ^]^ Furthermore, these devices show overall lower EQEs than panchromatic OPDs, which might be attributed to the lower charge carrier mobility and inappropriate BHJ morphology derived from strongly self‐assembling dyes. In order to further improve device performance, understanding the origin of the J‐type coupling is essential because optoelectronic properties can be significantly different depending on the interplay between short‐range (intermolecular charge transfer) and long‐range (Coulomb) couplings.^[^ ^11a,b^ ^]^

As a promising supramolecular building block, we have investigated dipolar dicyanovinylene‐substituted squaraine (SQ) dyes exhibiting favorable red to NIR absorption and emission properties already as monomers in solution.^[^ ^14^ ^]^ Due to the electron‐withdrawing dicyanovinylene group at the central squaric acid core, their optical transition from ground to the lowest singlet excited state (S_0_ → S_1_) is strongly bathochromically shifted, and accordingly this material class has shown potential in NIR applications including NIR emitters^[^ ^14^, ^15^ ^]^ and bioimaging.^[^ ^16^ ^]^ In addition to monomeric and aggregated states in solution, our comprehensive structure–property relationship study revealed that dipolar SQ can achieve high charge carrier mobility and strong excitonic coupling at the same time in thin‐film states.^[^ ^17^ ^]^ Although dipolar SQ thin films have shown their potential in optoelectronic devices such as organic solar cells,^[^ ^14^, ^18^ ^]^ photodetectors would be more suitable to fully utilize their interesting photophysical properties in their solid state.

**Figure 1:**
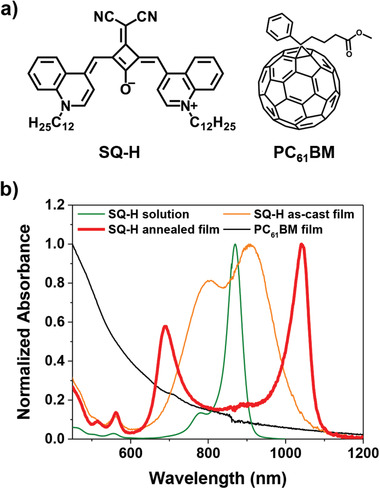
a) Chemical structures of the donor **SQ‐H** and the acceptor **PC_61_BM**. b) Normalized UV–vis–NIR absorption spectra of the **SQ‐H** in a 1 × 10^−5^ m CH_2_Cl_2_ solution (green solid line) and in spin‐cast thin film (orange solid line: as‐cast; red solid line: after annealing at 130 °C for 15 min) and of the **PC_61_BM** in thin‐film (black solid line) on quartz substrates.

In this work, we report a highly efficient SWIR organic photodetector based on the intermolecular charge transfer (ICT) mediated J‐type coupled squaraine dye **SQ‐H** (**Figure** **1a**). We explore the origin of the unique absorption spectra with a narrow absorption band at 1040 nm of **SQ‐H** thin films by optical, structural, and theoretical investigations. After optimizing BHJ thin films with **PC_61_BM**, we evaluate photodiode properties including spectral photoresponse, noise current, and response time of the devices. Due to a favorable combination of absorption and charge‐transport properties, the optimized BHJ OPDs provide maximum EQE value of 12.3% and FWHM of 85 nm (815 cm^−1^) at 1050 nm under 0 V. Based on the promising low‐power NIR photodiode property, we successfully applied the presented OPDs as a flexible heart‐monitoring sensor device.

## 2 Results and Discussion

**SQ‐H** shows unique optical properties in the solid state. Similar to typical absorption profiles of other squaraine dyes in amorphous thin films,^[^ ^19^ ^]^ an as‐cast **SQ‐H** layer exhibits a broad absorption spectrum with two distinguishable maxima at 909 and 799 nm, one of which is slightly redshifted and one slightly blueshifted with respect to its monomer in solution at 870 nm (Figure 1b, orange solid line). However, after an annealing process at 130 °C for 15 min, a full conversion from the amorphous to a well‐defined crystalline state is achieved, as indicated by two clearly distinguishable isosbestic points at 980 and 725 nm (Figure S2b, Supporting Information). The long‐wavelength absorption band (Figure 1b, red solid line) is significantly bathochromically shifted up to 1040 nm with an outstanding narrow FWHM of only 59 nm (555 cm^−1^). Interestingly, an additional new blueshifted absorption feature arises at 688 nm with approximately half the intensity of the NIR band and a FWHM of 65 nm (1335 cm^−1^). Along with a narrowband photoluminescence peak with little Stokes shift of only 90 cm^−1^ (Figure S2a, Supporting Information), these significant changes in the absorption profile of spin‐cast thin films during annealing indicate that strong intermolecular interactions occur upon aggregation, thereby enabling excitonic coupling between tightly packed chromophores.

**Figure 2:**
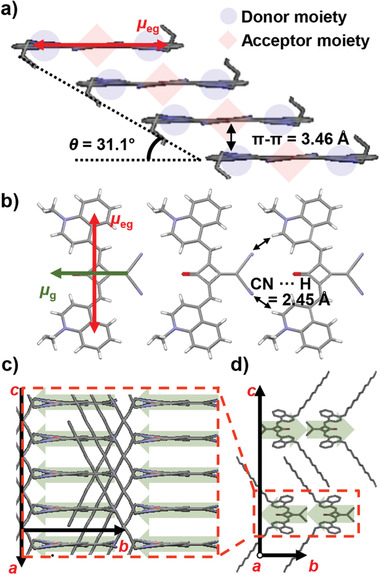
Packing arrangement of **SQ‐H** dyes in the single crystal. a) The slip‐stacked packing arrangement along the intrastack. The calculated S_0_ → S_1_ transition dipole moment (*µ*_eg_) is displayed as the red arrow. b) Interstack structure with CN···H intermolecular short contacts. The calculated ground state dipole moment (*µ*_g_) is displayed as the green arrow. c) Intralayer and d) interlayer structures of **SQ‐H** single crystal. The ground state dipole moments are schematically depicted behind the molecules as green shaded arrows.

Similar to previously reported dipolar SQ dyes,^[^ ^14^, ^17^ ^]^ time‐dependent density function theory (TD‐DFT) calculation of **SQ‐H** molecules demonstrate in accordance to early reports on dicyanovinylene substituted squaraine dyes that the ground‐state dipole moment (μ_g_) of about 13.7 D is oriented along the short molecular axis, whereas the S_0_ → S_1_ transition dipole moment (μ_eg_) is polarized along the long molecular axis. As revealed by the single‐crystal structure (**Figure** **2**),^[^ ^20^ ^]^ this ground‐state dipole moment, along with the donor–acceptor–donor type (D–A–D) molecular structure, plays an important role for the supramolecular organization in the solid state and the electronic coupling between the dyes.^[^ ^14^, ^17^ ^]^ Along the intrastack direction, the central dicyanovinylene substituted squaric acid acceptor moiety in one molecule interacts with donor moieties in the next neighboring molecules, resulting in a slip‐stacked molecular arrangement (θ = 31.1°) and tightly packed chromophores with a π–π distance of only 3.46 Å (Figure 2a). Obviously, significant intermolecular donor–acceptor overlap is present resulting from this slip‐stacked molecular arrangement.^[^ ^21^ ^]^ Interestingly, all *µ*_g_ are aligned parallel not only along the intrastack direction but also along the interstack direction, possibly due to the further stabilization by CN···H interactions between neighboring, head‐to‐tail oriented molecules in each layer (Figure 2b,c). Thus, the molecular ground‐state dipoles are surprisingly parallelly aligned in the separated layers parallel to the (001) crystal planes (Figure 2c), which is clearly distinguished from antiparallel cofacial stacking features of conventional dipolar SQ dyes.^[^ ^14^ ^]^ Consequently, along with interdigitating effects of the long C12 alkyl chains, an antiparallelly stacked layer‐by‐layer architecture is established to compensate the overall dipole moment of each subsequent layer (Figure 2d). Packing arrangements with these intermolecular interactions in a layered structure are indeed induced during the thermal annealing process, as can be monitored by UV–vis–NIR spectroscopy (Figure S2b, Supporting Information).

**Figure 3:**
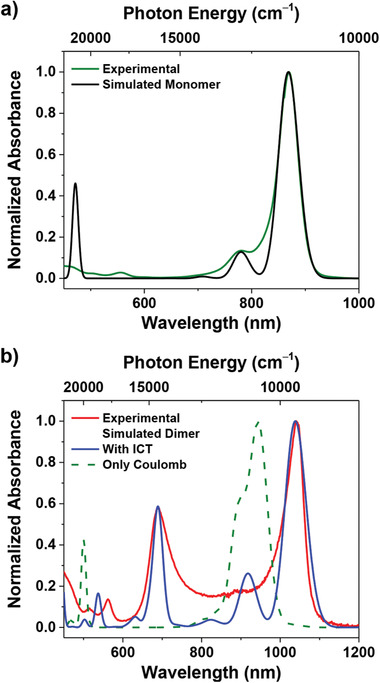
a,b) Simulated absorption profiles of **SQ‐H** as monomer (black solid line) (a) and as π‐stacked dimer in the single‐crystal structure (blue solid line: dimer with ICT coupling term; green dashed line: dimer without ICT coupling term) (b), along with the respective experimental absorption spectra in solution (a) and spin‐cast thin film (b).

In order to further elucidate the origin of the two absorption bands at 1040 and 688 nm of the **SQ‐H** thin films, theoretical calculations were conducted based on the packing structure found of the **SQ‐H** single crystal. First, the monomeric absorption spectrum was simulated in order to extract reliable parameters related to the neutral, zwitterionic, and vibrational states of the **SQ‐H**.^[^ ^22^ ^]^ As shown in **Figure** **3a**, the simulated monomeric absorption spectrum correlates well with the experimental spectral profile in dilute dichloromethane solution. Subsequently, Coulomb and ICT coupling terms were added to model the spectrum of a π‐stacked dimer unit extracted from the single‐crystal structure (Figure 2a) following the approach described in ref. ^[^ ^22^ ^]^. Comparing the simulated absorption spectra with and without ICT coupling term to the experimental spectrum, we conclude that the bathochromic shift of the NIR band (1040 nm) originates mainly from short‐range ICT (with a lesser contribution arising from the gas‐to‐crystal shift). Furthermore, it is obvious that the spectrum including only Coulomb coupling (Figure 3b, green dashed line) does not explain the band splitting, ruling out the presence of Davydov splitting. The absence of Davydov splitting is also obvious from the crystal structure revealing collinear alignment of the transition dipole moments of the two molecules within the unit cell (Figure 2). Moreover, the hypsochromically shifted absorption band (688 nm) in the annealed thin‐film is entirely attributable to ICT interactions resulting from pronounced donor–acceptor overlap between the stacked chromophores (Figure 3b; Figure S3, Supporting Information). Furthermore, the energetic separation between the low and high energy absorption band depends on the strength of the donor–acceptor overlap and not on the Coulomb coupling. All the parameters for the simulated absorption spectra are summarized in Table S2 in the Supporting Information.

The interesting optical features of **SQ‐H** are retained in **SQ‐H**:**PC_61_BM** BHJ thin films processed from 10 mg mL^−1^ chloroform solution. Figure S4 in the Supporting Information shows absorption spectra of **SQ‐H**:**PC_61_BM** blended thin films annealed at 90 °C. The characteristic two absorption bands in the red (688 nm) and NIR (1040 nm) spectral region are also found in annealed BHJ thin films, and the FWHM at red and NIR absorption spectral region are with 73 nm (1496 cm^−1^) and 71 nm (676 cm^−1^), respectively, almost retained in the two‐component layer. All the experimental optical characteristics are summarized in Table S3 in the Supporting Information. Although the FWHM values of BHJ thin films are slightly larger than those of **SQ‐H** single component thin films, this result indicates that the challenging requirements of λ > 1000 nm and FWHM < 100 nm for SWIR absorbing BHJ thin films can be achieved using the supramolecular J‐type aggregate engineering approach.

The pronounced ordering effect by the self‐assembly of dipolar **SQ‐H** can have a great impact on charge transport characteristics in neat **SQ‐H** as well as BHJ thin films as previously reported for similar chromophores.^[^ ^17^ ^]^ In order to evaluate the charge‐transport properties of the **SQ‐H** thin films, organic thin‐film transistors (OTFTs) and single charge carrier devices (i.e., hole‐ and electron‐only diodes) were fabricated. As expected from the well‐ordered two‐dimensional layered structure and high crystallinity, spin‐coated OTFTs of **SQ‐H** exhibited decent field‐effect hole mobility of 1.4 × 10^−2^ cm^2^ V^−1^ s^−1^ on hexamethyldisilazane treated Si/SiO_2_ substrates in the saturation regime (Figure S5 and Table S4, Supporting Information). The vertical charge carrier mobilities of **SQ‐H**:**PC_61_BM** BHJ thin films were measured using the space charge limited current (SCLC) model at varying annealing temperatures (Figure S6, Supporting Information).^[^ ^23^ ^]^ The calculated SCLC hole/electron mobilities of the BHJ devices were 4 × 10^−5^/9 × 10^−7^, 1 × 10^−3^/2 × 10^−5^, and 2 × 10^−3^/7 × 10^−4^ cm^2^ V^−1^ s^−1^ for as‐cast, 90, and 130 °C annealing conditions, respectively. The increasing mobility values, particularly a huge improvement of the hole mobility, with increased annealing temperature imply that the self‐assembled **SQ‐H** nanostructure has a positive effect on the overall device performance. To better understand the origin of beneficial annealing effects for charge transport, selected area electron diffraction (SAED) and out‐of‐plane X‐ray diffraction (XRD) experiments were conducted for thin films. The SAED from **SQ‐H** single‐crystalline domain exhibited the interplanar distance (*d*‐spacing) values of 0.81, 0.68, and 0.52 nm, which are correlated to the (012), (100), and (112) plane in the single‐crystal structure, respectively (Figure S7a–c, Supporting Information). From this assignment, we can assume that the *c*‐axis of the **SQ‐H** thin film structure is tilted by about 30° with respect to the substrate surface normal, resulting in *d*‐spacing of ≈3 nm along the out‐of‐plane direction (Figure S7d, Supporting Information). Indeed, as shown in Figure S8 in the Supporting Information, the XRD pattern in annealed **SQ‐H** single component thin films showed a reflex at 3.01° (*d*‐spacing = 2.94 nm). The SAED patterns in polycrystalline neat **SQ‐H** exhibited an additional *d*‐spacing value of 0.96 nm corresponding to (010) plane, indicating that there are crystallites with a vertically oriented *c*‐axis respected to substrate surface (Figure S9a, Supporting Information). However, because of the systematically extinguished nature, no diffraction pattern was observed corresponding to (001) plane from all the thin film XRD experiments. The SAED patterns of BHJ thin films with **PC_61_BM** displayed *d*‐spacing value of around 0.81 nm corresponding to (012) plane, which indicates that **SQ‐H** structure in BHJ thin films is analogous to that in the neat SQ‐H thin film (Figure S9b–e, Supporting Information). Similar to the neat **SQ‐H** XRD pattern, BHJ thin films show only one XRD reflex at 3.08° (*d*‐spacing = 2.86 nm), exhibiting increasing intensity for elevated annealing temperatures, which is indicative for a beneficial contribution of the tilted nanoscale crystallization of **SQ‐H** to the charge carrier mobility. This nanoscale crystallization also changes the morphology of BHJ thin films depending on annealing temperatures. To find the optimum nanomorphology for **SQ‐H**:**PC_61_BM** active layers, we investigated the effect of the annealing temperature on the film morphology by atomic force microscopy (AFM) and scanning electron microscopy (SEM). According to our AFM and SEM studies (Figure S10, Supporting Information), OPDs annealed at 90 °C showed the best performance (vide supra; Figure S11, Supporting Information).

**Figure 4:**
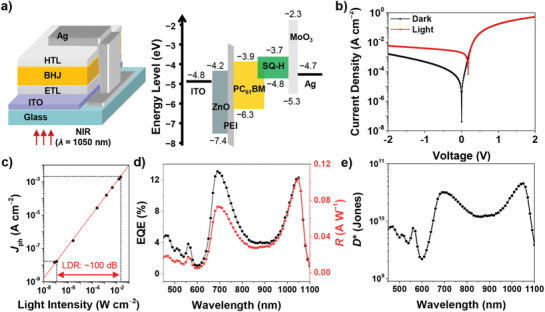
a) Schematic device architecture and energy diagram of the **SQ‐H**:**PC_61_BM** blend‐based OPDs. b) Representative *J*–*V* curves of the OPD in the dark (black line) and under illumination (red line) with NIR light (≈20 mW cm^−2^@1050 nm). c) Linear dynamic range of the OPDs under selective illumination at 1050 nm at 0 V. d) EQE (black) and responsivity *R* (red) as well as e) specific detectivity D* of OPDs at 0 V.

Based on the optical, electrical, and morphological analyses of the optimized active layer with a thickness of about 85 ± 6 nm, OPDs were fabricated in an inverted device architecture: glass/indium tin oxide(ITO)/zinc oxide (ZnO)/polyethyleneimine (PEI)/**SQ‐H**:**PC_61_BM** (1:1.5 weight ratio)/molybdenum oxide (MoO_3_)/Ag (**Figure** **4a**; the detailed fabrication procedure is described in the Experimental Section). The energy levels of **SQ‐H** and **PC_61_BM** were calculated from the redox potential obtained by cyclic voltammetry in CH_2_Cl_2_ solution (Figure S12, Supporting Information). The current density (*J*)–voltage (*V*) characteristics under 1050 and 680 nm illumination were evaluated (Figure S13, Supporting Information). Figure 4b shows the *J*–*V* characteristics in the dark and under selective 1050 nm illumination (≈20 mW cm^−2^). The OPDs showed sufficiently low dark current density of ≈40 nA cm^−2^ at short‐circuit conditions (0 V), which is beneficial for detecting weak light signals without additional power consumption. Under the illumination with 1050 nm NIR light of ≈20 mW cm^−2^, the photocurrent density reaches up to ≈2 mA cm^−2^ at 0 V condition, indicating that our device is highly sensitive to NIR light. With decreasing light intensity, the photocurrent density (*J*_ph_, the current density difference between illumination and dark conditions) gradually decreased as shown in Figure 4c and Figure S14 in the Supporting Information. This characteristic can be evaluated by the linear dynamic range (LDR) value, which represents the range in which the input light power and output signal show a linear relationship. The LDR can be calculated by

(1) $$\begin{array}{c} \text{LDR}\;=\;20\log\frac{J_{\max}}{J_{\min}} \end{array}$$

where *J*_max_ and *J*_min_ are the maximum and minimum detectable photocurrent density, respectively, before deviating from the linear relationship. According to Equation (1), we estimated an LDR value of ≈100 dB, which is higher than that of an inorganic NIR photodetector based on InGaAs (66 dB).^[^ ^24^ ^]^

In order to investigate the spectral photoresponse and corresponding responsivity, EQE spectra were recorded from 450 to 1100 nm in steps of 10 nm. Figure 4d displays the EQE value of 12.3% at 1050 nm, which is one of the highest EQE values among the NIR OPDs exhibiting maximum EQE peak over 1000 nm without sample bias voltage. In addition, an EQE value of 13.1% at 690 nm was also observed. The EQE spectra closely follow the spectral profile of the BHJ thin films, and thus the FWHM values at 1050 and 690 nm reach an optimum of 85 nm (815 cm^−1^) and 113 nm (2227 cm^−1^), respectively. Note that due to limitations of the experimental setup, we could only record EQE spectra in 10 nm steps, meaning the actual FWHM at 1050 nm from our OPD could be smaller than 85 nm and the EQE even higher than 12.3%. To the best of our knowledge, this is the first NIR OPD exhibiting a FWHM < 100 nm with an EQE > 10% in SWIR, which simply originates from the exchange narrowing of the **SQ‐H** chromophores without the additional need of optically fine‐tuning of the device architecture.

The responsivity (*R*) is an important figure‐of‐merit for evaluating photodetectors, which can be calculated from EQE values by

(2) $$\begin{array}{c} R\;=\frac{\text{EQE}}{100\%}\;\times \frac{\lambda_{\text{input}}}{1240\;\left(\text{nm}\;W\;A^{-1}\right)} \end{array}$$

where λ_input_ is the incident light wavelength. Accordingly, *R* is 0.1 A W^−1^ under illumination with 1050 nm at 0 V (Figure 4d), which is well consistent with *R* from *J*–*V* characteristics (Figure S14b, Supporting Information). By applying a reverse bias of −2 V, the EQE and *R* at 1050 nm are increased, reaching up to 24.1% and 0.2 A W^−1^, respectively (Figure S15a,b and Table S5, Supporting Information).

Based on the EQE and *R*, the specific detectivity (*D* ^*^) was evaluated. The specific detectivity can be expressed by

(3) $$\begin{array}{c} D^{*}=\frac{R\sqrt{A\Delta f}}{i_{\text{noise}}} \end{array}$$

where *A* is the area of the active layer (7.1 mm^2^), Δ*f* is the bandwidth, and *i*_noise_ is the noise current. In OPDs, the shot noise (*i*_shot_) and thermal noise (*i*_thermal_) are major components of the noise current. Accordingly, the noise current can be estimated by^[^ ^25^ ^]^

(4) $$\begin{array}{c} i_{\text{noise}}\approx \;\sqrt{{\left(i_{\text{shot}}\right)}^{2}+{\left(i_{\text{thermal}}\right)}^{2}}=\sqrt{{\left(2qI_{\text{dark}}\Delta f\right)}^{2}+{\left(\frac{4k_{B}T\Delta f}{R_{\text{shunt}}}\right)}^{2}} \end{array}$$

where *q* is the elementary charge, *I*_dark_ is the dark current, *k*_B_ is the Boltzmann constant, *T* is the temperature, and *R*_shunt_ is the shunt resistance. From the current–voltage characteristics, the noise current was estimated to 6 × 10^−13^ A Hz^−0.5^ at 0 V, where thermal noise dominated over shot noise (Figure S16a, Supporting Information). However, as reverse bias increased, shot noise became the main component of the noise current due to the rapid increase in dark current. Consequently, in the reverse bias condition, the detectivity decreased despite the increase in EQE and responsivity (Figure S15c and Table S5, Supporting Information). Thus, **SQ‐H** OPD exhibited a maximum detectivity of 4 × 10^10^ Jones at 1050 nm under short‐circuit condition. To avoid an overestimation of detectivity level,^[^ ^25^ ^]^ the actual noise spectral density was directly recorded by a lock‐in amplifier (Figure S16b, Supporting Information). Above 30 Hz, the noise current fluctuated between 1 × 10^−12^ and 3 × 10^−13^ A Hz^−0.5^. Accordingly, the experimental noise current gave detectivity levels between 2 × 10^10^ and 9 × 10^10^ Jones, which agreed well with the calculated detectivity value from *J*–*V* characteristics.

To evaluate the response speed of the SWIR OPDs, the transient photoresponse was investigated under frequency‐modulated NIR illumination at 1050 nm and 0 V sample bias. As shown in Figure S17a in the Supporting Information, the photoresponse from the device can catch up with the 10 kHz modulated light input, providing a steady‐state on/off response signal. However, at a modulation of 300 kHz, the photoresponse is not adequate and the saturation level/amplitude is not reached (Figure S17b, Supporting Information). Regarding the abovementioned characteristics, the cut‐off frequency is an important parameter, which is defined as the frequency at which the output signal from a photodetector is reduced to −3 dB (70.8% of the original amplitude). As displayed in Figure S17c in the Supporting Information, the cut‐off frequency is determined to be around 300 kHz, which is sufficient for low‐power operating wearable health monitoring applications. Considering the estimated transit time‐limited cutoff frequency (*f*_tr_ = 330 kHz) and the RC time constant‐limited cutoff frequency (600 kHz < *f*_RC_ < 1.2 MHz), we assume that photoresponse of **SQ‐H**‐based OPDs is limited by a relatively low electron mobility of **PC_61_BM** (the details of calculation are described in Figure S17 in the Supporting Information).

**Figure 5:**
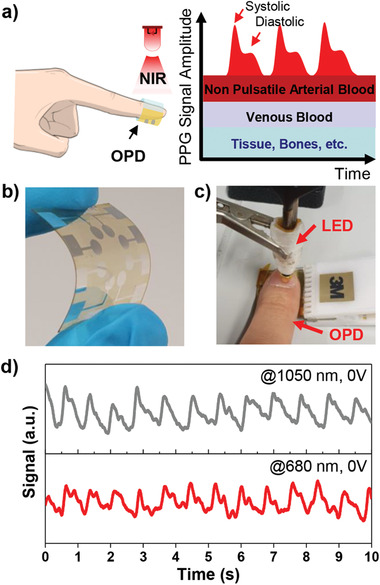
a) Schematic representation of the measuring setup of **SQ‐H‐** based OPD devices to record a photoplethysmogram by LED illumination at 1050 and 680 nm in transmission mode. b,c) Photographs of a flexible OPD (b) and the photoplethysmogram sensor demonstration using the **SQ‐H**‐based device (c). d) Pulse signal measured from the OPD under 1050 and 680 nm LED illumination through the fingertip.

In order to demonstrate the applicability for practical wearable electronics, transmission mode photoplethysmography (PPG) measurements were conducted with flexible **SQ‐H** OPDs. The **SQ‐H** BHJ OPDs on flexible substrates exhibited slightly lower performance of 9% EQE at both 690 and 1050 nm (Figure S18, Supporting Information). A schematic PPG setup and the photoresponse from OPDs for red and NIR light passing through human tissue are shown in **Figure** **5**. Thus, we can extract clear periodic signals including systolic and diastolic points with our OPD for both wavelengths under ambient conditions (Figure 5d). As expected due to the better penetration depth in human tissue, the signals for 1050 nm illumination outperform the ones for 680 nm. From the average interval between systolic peaks from signals under 1050 nm LED, we can calculate the heart rate of 74 beats min^−1^. Because red and NIR wavelengths are commonly used for transmission mode oximeter applications due to their response to oxygenated hemoglobin (stronger absorption of NIR light) and deoxyhemoglobin (stronger absorption of red light), we assume that our OPDs may also be optimized for recording the blood oxygen saturation level.^[^ ^26^ ^]^

## 3 Conclusion

We have demonstrated an efficient narrowband BHJ OPD based on a dicyanovinylene functionalized squaraine dye (**SQ‐H**) as donor material for NIR sensor applications. The annealed **SQ‐H** thin‐film exhibited narrow absorption bands in the red (λ = 688 nm, FWHM = 65 nm (1335 cm^−1^)) and NIR (λ = 1040 nm, FWHM = 59 nm (555 cm^−1^)) region, which originate from ICT‐mediated coupling. In addition to the unique optical properties, favorable nanomorphology could be induced by thermal annealing, leading to charge carrier mobility enhancement in **SQ‐H**:**PC_61_BM** BHJ thin films. The optimized OPDs exhibited EQEs of up to 12.3% and FWHM of 85 nm (815 cm^−1^) at 1050 nm under short‐circuit conditions (0 V), which is beneficial for low‐power consuming practical applications. Indeed, we successfully demonstrated a heart‐rate monitoring PPG application with the flexible OPDs upon selective excitation through tissue at 1050 nm. To the best of our knowledge, these are the first OPDs showing narrow spectral response (FWHM < 100 nm) with an EQE over 10% in SWIR region (λ > 1000 nm). The simplicity of the supramolecular engineering approach for the creation of intense NIR bands with pronounced exchange narrowing by J‐type aggregation of organic semiconductors is clearly advantageous for high‐performing color selective NIR OPDs compared to previous approaches that depend on complex device architectures.

## Conflict of Interest

The authors declare no conflict of interest.

## Supporting information

Supporting Information

## Data Availability

Research data are not shared.
