# Supplementary material for: An Efficient Narrowband Near‐Infrared at 1040 nm Organic Photodetector Realized by Intermolecular Charge Transfer Mediated Coupling Based on a Squaraine Dye
Source: Adv Mater. 2021 May 31;33(26):2100582. doi: 10.1002/adma.202100582 (PMC11469076; doi:10.1002/adma.202100582)
Supplement: Supplementary file 1 — Supporting Information [file ADMA-33-2100582-s001.pdf]

© 2021 Wiley-VCH GmbH

ADVANCED MATERIALS

Supporting Information

for *Adv. Mater.,* DOI: 10.1002/adma.202100582

An Efficient Narrowband Near-Infrared at 1040 nm Organic Photodetector Realized by Intermolecular Charge Transfer Mediated Coupling Based on a Squaraine Dye

*Jin Hong Kim, Andreas Liess, Matthias Stolte, Ana-Maria Krause, Vladimir Stepanenko, Chuwei Zhong, David Bialas, Frank Spano, and Frank Würthner**

WILEY-VCH

Copyright 2021 Wiley-VCH GmbH

Supporting Information

An Efficient Narrowband Near-Infrared at 1040 nm Organic Photodetector Realized by Intermolecular Charge Transfer Mediated Coupling Based on a Squaraine Dye

*Jin Hong Kim, Andreas Liess, Matthias Stolte, Ana-Maria Krause, Vladimir Stepanenko, Chuwei Zhong, David Bialas, Frank Spano, and Frank Würthner**

Table of Contents

Experimental Section

Supporting Figures and Discussion: Figure S1-S18

Supporting Tables: Table S1-S6

Supporting References

Experimental Section

Materials: All materials were purchased from commercial suppliers and used without further purification. **SQ-H** was prepared by the previously reported synthetic procedure.[S1] Cyclic voltammetry measurements were performed on a standard commercial electrochemical analyzer (EC epsilon, BAS Instruments) in a three-electrode single-compartment cell under argon (working electrode: Pt disc, reference electrode: Ag/AgCl, and counter electrode: Pt wire). The measurements were carried out in CH_2Cl_2 with a 0.1 M tetrabutylammonium hexafluorophosphate (TBAHPF) solution.

Optical property: UV-Vis-NIR absorption spectra were recorded by a Perkin Elmer Lambda 950 UV-Vis-NIR spectrometer equipped with an integration sphere. The **SQ-H** thin films were prepared by spin-coating a solution of **SQ-H** (2 mg mL^{-1} $CHCl_3$) on a quartz substrate. Thermal annealing was performed with a Gestigkeit PZ28-2 precision heating stage as indicted in the manuscript. Fluorescence spectra of annealed thin films were measured at room temperature on an Edinburgh Instruments FLS980-D2D2-ST spectrometer with a front face sample holder F-J03 (22.5°) and were corrected against the photomultiplier sensitivity and the lamp intensity. For **SQ-H**:**$PC_{61}BM$** BHJ thin films, the donor-acceptor blend solution of **SQ-H**:**$PC_{61}BM$** (1:1.5 weight ratio) in $CHCl_3$ with a concentration of 10 mg mL^{-1} was spin-coated on quartz substrates, followed by an annealing process for 10 min at different temperatures.

Theoretical calculations: For estimating ground-state dipole moment and transition dipole moment, geometry optimization and time-dependent density functional theory calculation were carried out using B3LYP functional and 6-311G (d,p) basis sets as implemented Gaussian16 Package.[S2] All the theoretical calculations and simulations related to the optical properties were conducted according to the previously reported methods.[S3] The effective charge transfer

integrals were calculated with PW91 functional and TZP basis sets using Amsterdam Density Functional package.[S4] The nearest-neighbor dimer geometries were directly adopted from single crystal structure.

X-ray diffraction: Single crystal X-ray diffraction data for compound **SQ-H** were collected at ambient temperature (300 K) on a Bruker D8 Quest Kappa Diffractometer with a Photon100 CMOS detector and multi-layered mirror monochromated $Cu_{K\alpha}$ radiation. The solved structure was obtained with Fourier techniques and the Shelx software package.[S5] Crystallographic data are deposited with the Cambridge Crystallographic Data Centre as supplementary publication no. CCDC 2057832 (**SQ-H**). For out-of-plane XRD, **SQ-H** and **SQ-H**:**$PC_{61}BM$** (1:1.5 weight ratio) chloroform solutions (20 mg mL^{-1}) were spin-coated on zinc oxide (ZnO) deposited indium-tin oxide (ITO) substrates. And then, out-of-plane XRD were measured by a Bruker D8 Discover diffractometer with a LynxEye-1D-Detecter and $Cu_{K\alpha}$ radiation.

Selected area electron diffraction (SAED): For thin-film SAED measurements, **SQ-H** chloroform solutions (1 mg mL^{-1}) and **SQ-H**:**$PC_{61}BM$** (1:1.5 weight ratio) chloroform solutions (10 mg mL^{-1}) were spin-coated on PEDOT:PSS coated glass substrates, and then thin films were annealed at different temperature for 10 min. Then the substrates were immersed in purified water and the floating thin films were transferred from water to carbon-coated copper grids (Agar Scientific Ltd., Lacey Carbon Films on 200 Mesh Copper Grids). Afterwards, the SAED measurements were carried out using a FEI Titan 80-300 transmission electron microscope with an accelerating voltage of 150 kV.

Morphology Characterization: AFM measurements were performed under ambient conditions using a Bruker Multimode 8 SPM system operating tapping mode. Silica cantilevers (OMCL-AC200TS, Olympus) with a resonance frequency of ~150 kHz and a spring constant of ~9 N m^{-1} were used. SEM images were recorded using a Carl Zeiss Ultra plus field emission scanning electron microscope equipped with GEMINI e-beam column operated at 1.5 kV with an aperture size set to 30 μm to avoid excessive charging and radiation damage of the areas imaged. The thin films for AFM and SEM measurement were prepared by spin-coating method (**SQ-H**:**$PC_{61}BM$**=1:1.5 weight ratio, 20 mg mL^{-1} in chloroform, 2000 rpm, 60 s) on the Si/SiO_2 substrates.

Transistor Devices Fabrication: The **SQ-H** thin films were deposited on the hexamethyldisilazane (HMDS) treated Si/SiO_2 substrates by spin-coating process (2 mg mL^{-1} chloroform, 5000 rpm for 30s), and then the thin films were annealed at 130 ºC for 15 min. Au was deposited on the **SQ-H** thin films for source and drain electrodes.

Diode Devices Fabrication: Prior to fabrication ITO coated glass substrates were cleaned with detergent, and sonicated in distilled water, acetone and isopropyl alcohol for 10 min. After drying the substrates by N_2 stream, UV-ozone treatment was carried out for 30 min. For the ZnO layer on the cleaned substrates, the ZnO precursor solution was prepared using a mixture of zinc acetate (400 mg), 2-methoxyethanol (4 mL), and ethanolamine (715 μL). Then a ZnO film was deposited by spin-coating at 2000 rpm for 60s and annealed at 150 ℃ for 10 min in air. Polyethyleneimine (PEI, Mw = 25,000, Sigma-Aldrich) was dissolved into 2-methoxyethanol with 0.05 wt% and then PEI solution was spin-coated onto the substrates at 5000 rpm for 30s followed by an annealing process at 110 ℃ for 10 min in air. To form the bulk-heterojunction active layer (~ 85 nm), a blend solution of **SQ-H**:**$PC_{61}BM$** (1:1.5 weight ratio) in chloroform (20 mg mL^{-1}) was spin-coated on top of the ZnO layer under inert

conditions (M. Braun Inertgas Systeme GmbH; $c(O_2) < 0.1$ ppm, $c(H_2O) < 0.1$ ppm). After transferring to the OPVIvap-XL (Creaphys GmbH) vacuum chamber (pressure of ca. 10^{-7} mbar), MoO_3 (10 nm) and Ag (100 nm) electrodes were deposited on the active layer by thermal evaporation with an active area of 7.1 mm^2. Finally, post annealing process was carried out on a Gestigkeit PZ28-2 precision heating stage at different temperatures for 10 min. To make the hole-only diodes, 30 mg mL^{-1} of **SQ-H**:**PC$_{61}$BM** (1:1.5 weight ratio) blend solution in chloroform was spin-coated on the MoO_3 (10 nm) deposited ITO substrates and then MoO_3 (10 nm) and Ag (100 nm) were subsequently deposited for the top electrode. For the electron-only diodes, the blend solution was spin-coated on the ZnO deposited ITO substrates, followed by Ca (6 nm) and Al (100 nm) thermal evaporation. Using the ITO coated polyethylene terephthalate (PET) films (Sigma-Aldrich, 639303), the flexible devices were fabricated by the same procedure as for the optimized rigid OPDs.

Device Characterization: The current density vs. voltage (*J-V*) characteristics were measured using an Agilent 4155C Semiconductor Parameter Analyzer in dark and under irradiation of monochromic light under inert conditions (M. Braun Inertgas Systeme GmbH; $c(O_2) < 0.1$ ppm, $c(H_2O) < 0.1$ ppm). For illuminating monochromic light, light-emitting diodes (Thorlabs, LED1050L and LED680L) with a peak wavelength of 1050 nm and 680 nm were used. The light intensity was measured by a powermeter (Newport, 2832-C) equipped with a calibrated silicon photodiode (Newport, 818-SL). The external quantum efficiency (EQE) was measured using a Newport QE/IPCE Measurement Kit comprised of a 300 W xenon lamp, a monochromator (Newport, 74125), a Merlin lock-in amplifier (Newport, 70104), an optical chopper, and a calibrated silicon photodiode (Newport, 70356_70316NS). All of the EQE were measured under a monochromatic light source modulated at 30 Hz using the optical chopper. For measurement of transient response, the LED light source (Thorlabs, LED1050L) was

modulated by a built-in waveform generator module (Agilent, DSOX2WAVEGEN) of an oscilloscope (Agilent, DSO-X 2002A) for triggering the photoresponse of OPDs. Then photoresponse signals were recorded by an oscilloscope (Agilent, DSO-X 2002A) with a 50 Ω load resistor. The noise current was directly measured by using a lock-in amplifier (Signal Recovery, 7265). For photoplethysmography (PPG) measurement, the output of the OPDs were amplified by a low noise preamplifier (PerkinElmer instruments, Model 5182) with a A/V gain factor of 10^{-7}, and then recorded by an oscilloscope (Agilent, DSO-X 2002A) with a low-pass electrical filter (Thorlabs, EF508). The PPG was collected from one of the authors under their informed consent. No ethical approval was necessary.

Supporting Figures

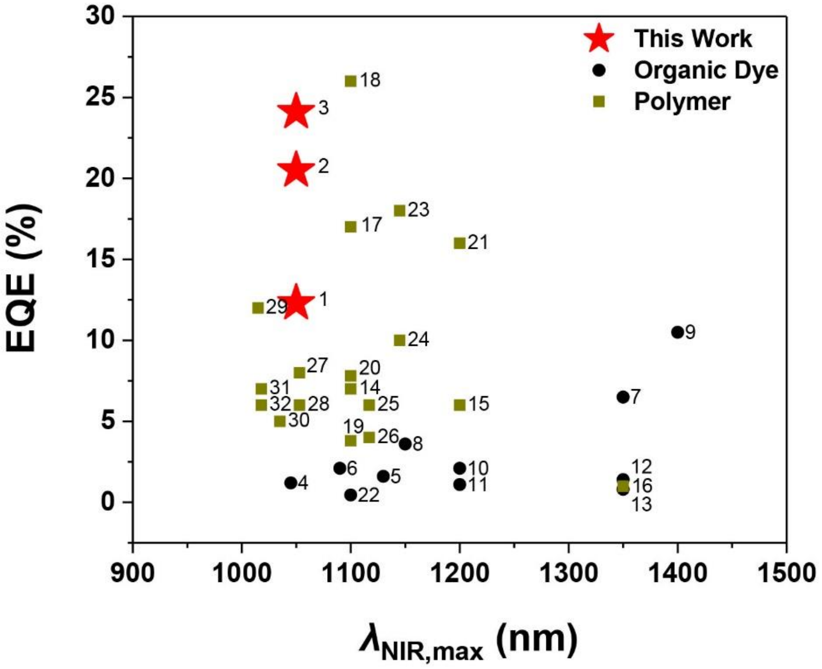

Figure S1. External quantum efficiency (EQE) as function of maximum wavelength at NIR region ($\lambda_{NIR,max}$) accomplished in this work in comparison to previously reported organic dye and polymer OPDs (see also Table S1).

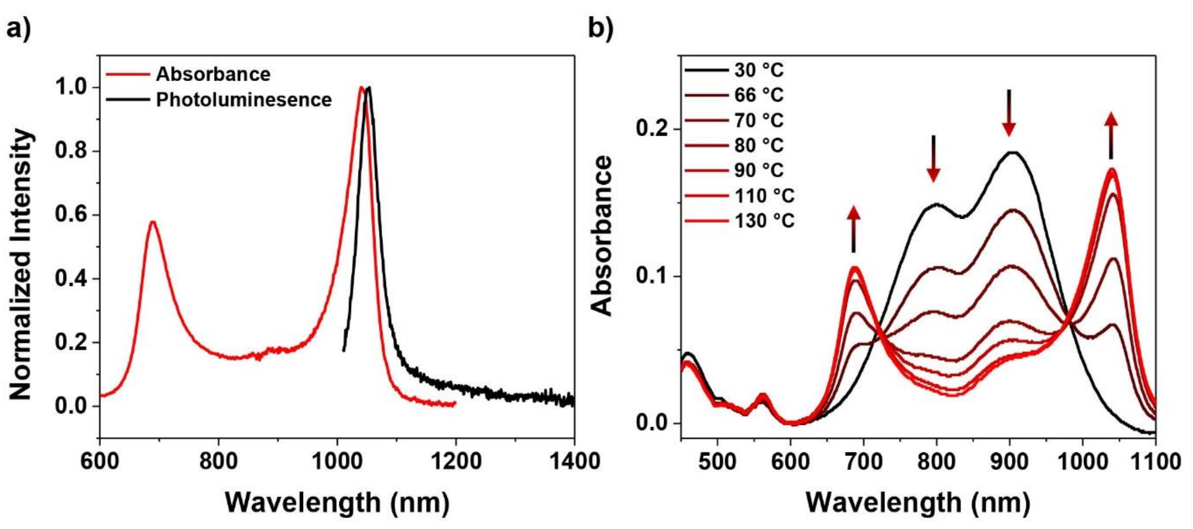

Figure S2. Optical properties of **SQ-H** thin-film. a) Normalized UV-Vis-NIR absorption and fluorescence (λ_{ex} = 945 nm) spectra of spin-coated and annealed thin-film of **SQ-H** on quartz. b) Change in UV-Vis-NIR absorption spectra of **SQ-H** on quartz recorded in-situ during annealing process with a rate of 2 K min^{-1}.

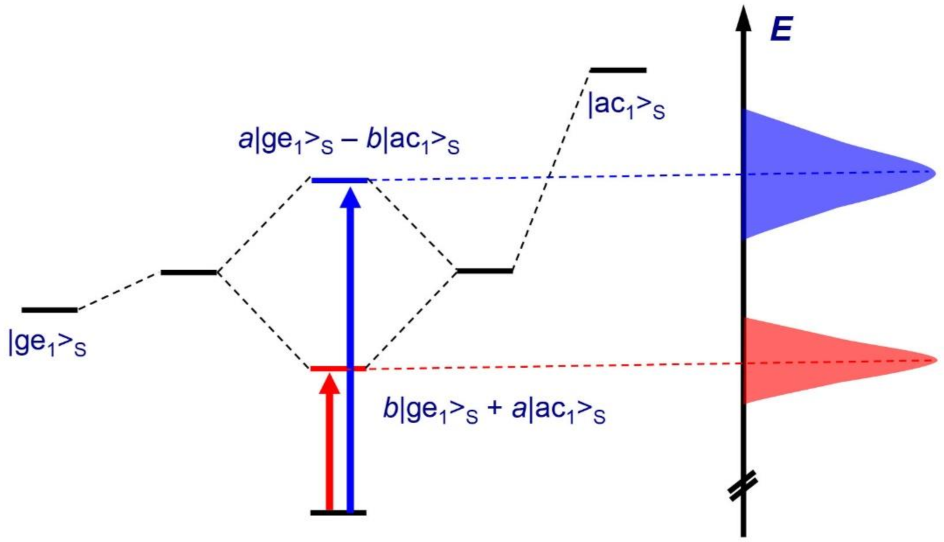

Figure S3. Schematic energy diagram for squaraine dimer. $|ge_1>_S$ represents the optically allowed state of the non-interacting dimer, whereas $|ac_1>_S$ is a symmetric charge separated state (for details see Ref. S3). The symmetry is defined with regard to translation. The destabilization and stabilization of states $|ge_1>_S$ and $|ac_1>_S$, respectively, arises from Coulomb interaction between the chromophores. Accordingly, the two states are brought into resonance and are coupled leading to a splitting with two optically allowed states, which explains the two-band structure observed in the UV-Vis-NIR absorption spectrum of **SQ-H** in thin film.

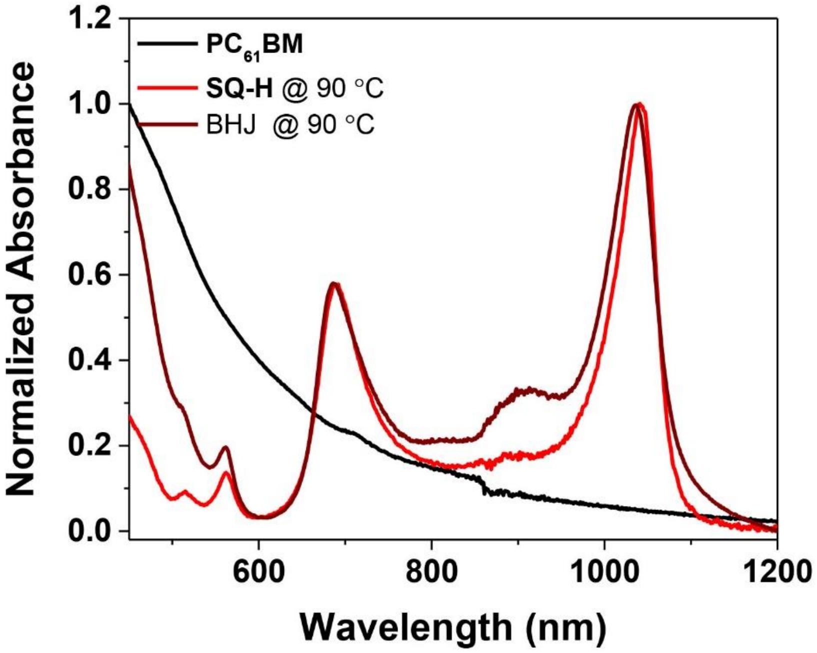

Figure S4. Scaled absorption spectra of the **SQ-H**, **$PC_{61}BM$,** and bulk-heterojunction thin films thereof after thermal annealing.

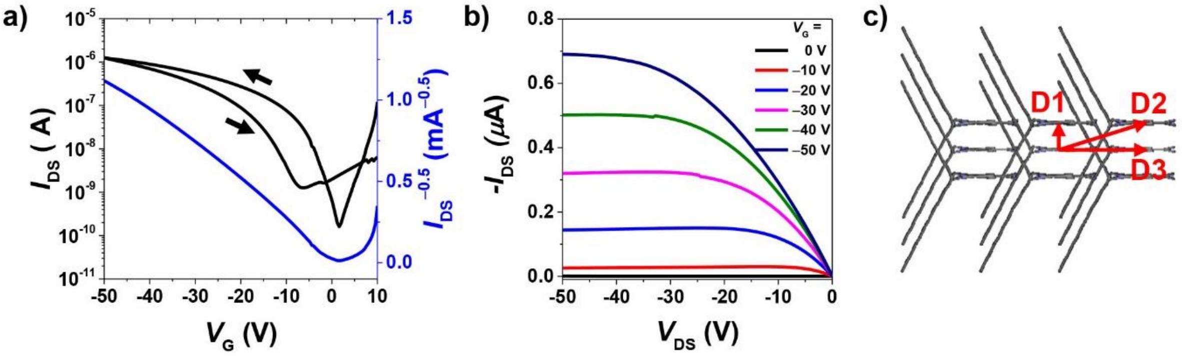

Figure S5. a) Transfer and b) output characteristics of organic field-effect transistors of neat **SQ-H** thin films on Si/SiO_2/HDMS substrates after thermal annealing. c) The nearest-neighbor dimers along the in-plane of the **SQ-H** molecular packing structure (see also Table S4).

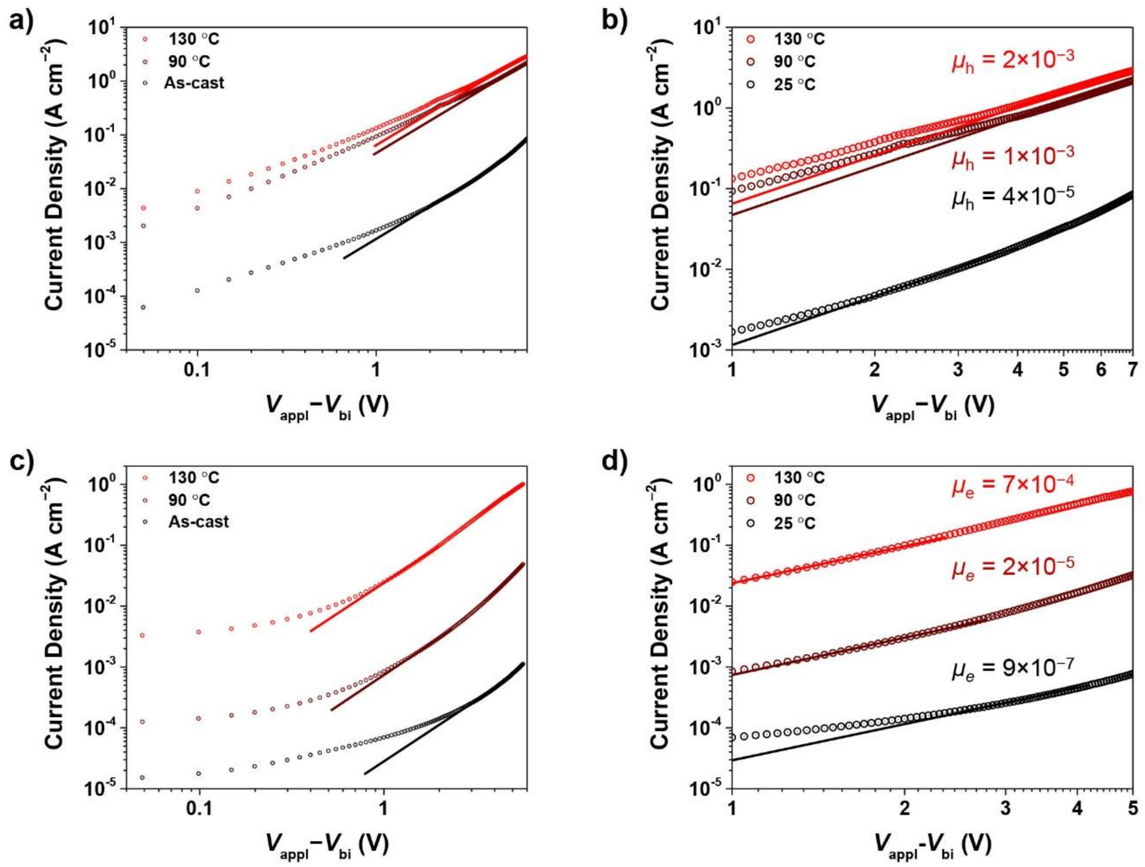

Figure S6. *J*-*V* characteristics of the a,b) hole- and c,d) electron-only **SQ-H**:**PC$_{61}$BM** bulk-heterojunction devices under different annealing conditions. The SCLC mobility values are denoted in the figures. μ_h and μ_e represent hole and electron SCLC mobility, respectively.

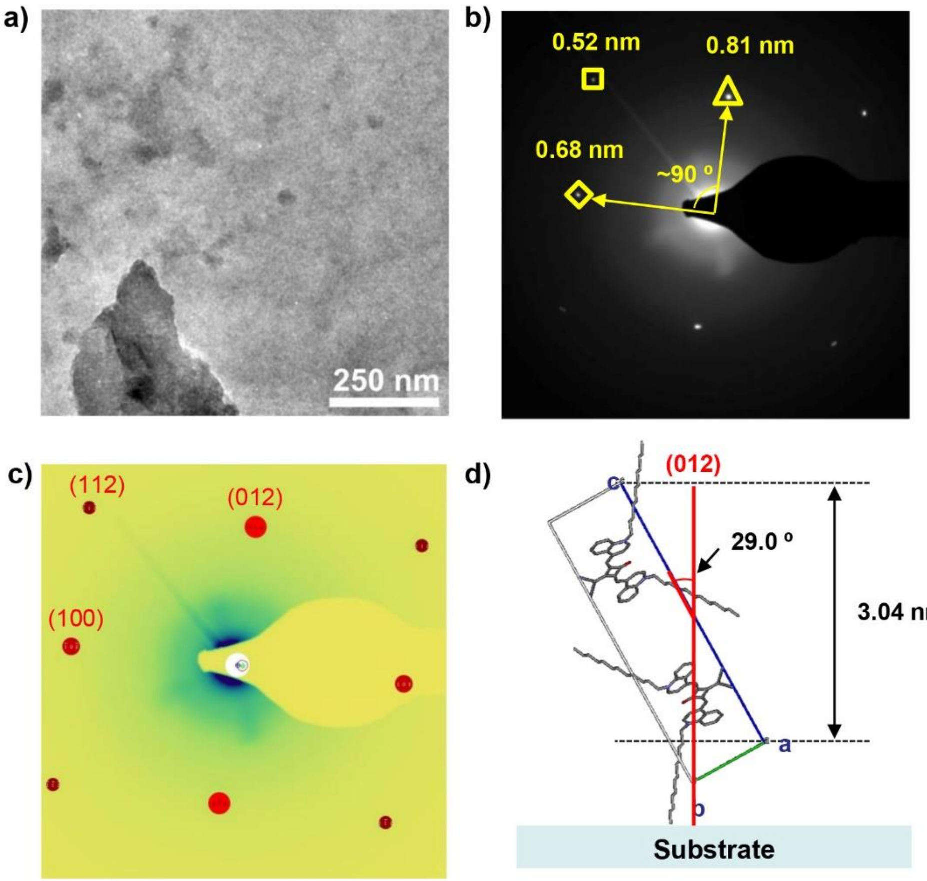

Figure S7. a) TEM image of the neat **SQ-H** thin film annealed at 150 °C for 10 min. b-c) The corresponding SAED patterns and their respective simulation of single crystalline **SQ-H** domain indexed as (100), (012), and (112) planes. d) Schematic of the tilted **SQ-H** thin film structure. During the SAED recordings, the organic thin films (bottom left dark region in TEM image) were damaged.

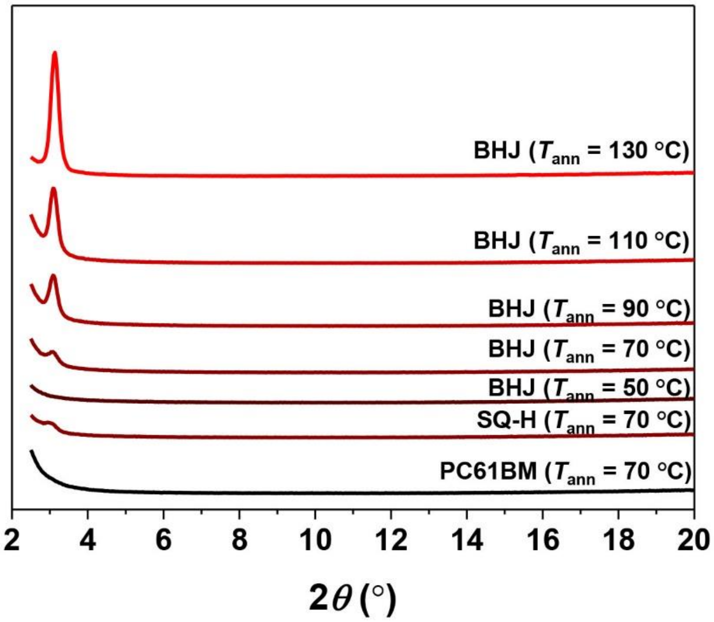

Figure S8. Out-of-plane XRD patterns for the **SQ-H** (dark red) **$PC_{61}BM$** (black) and their bulk-heterojunction (colored) thin films on ZnO deposited ITO substrates. The respective annealing temperatures (T_{ann}) are denoted in the figure.

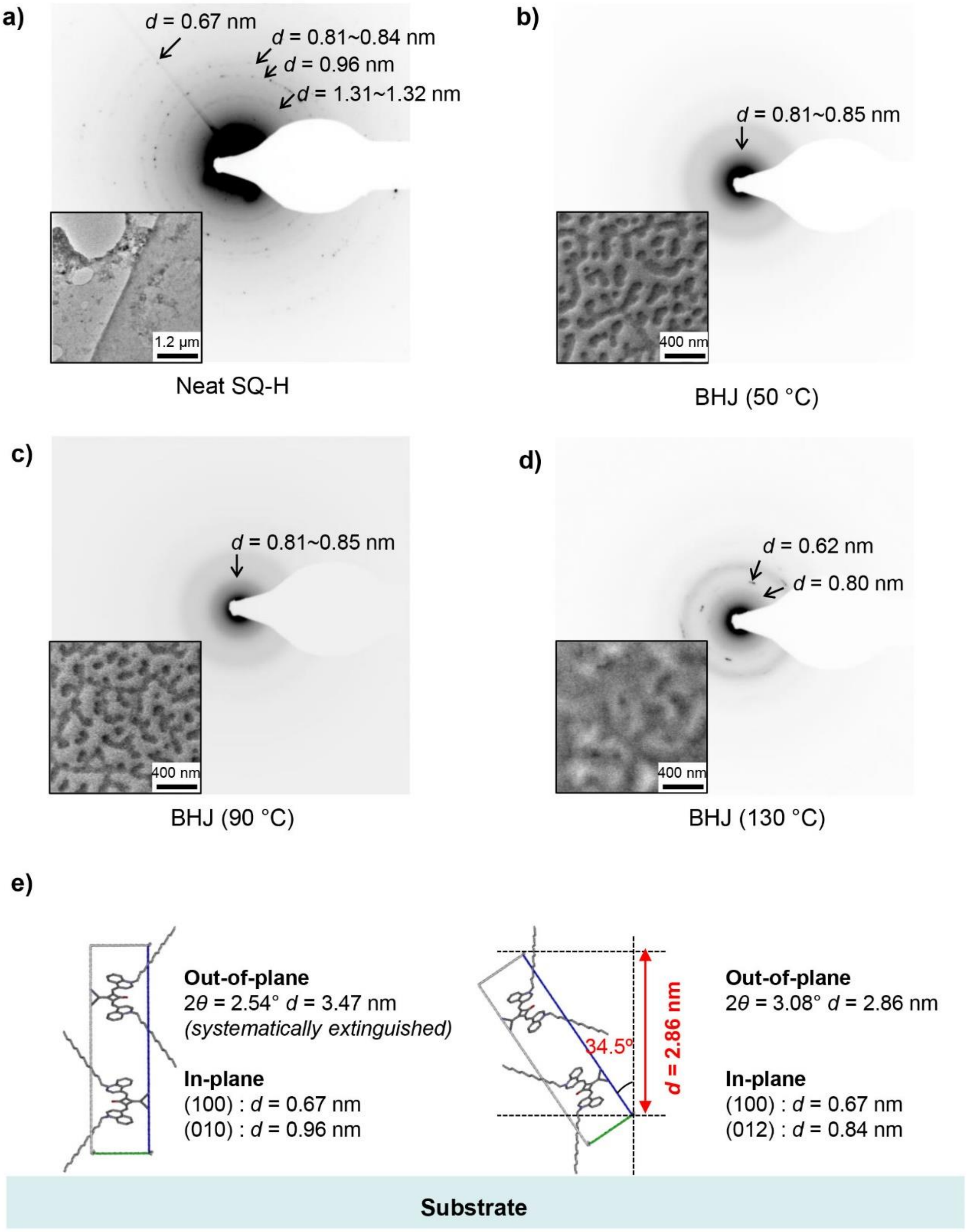

Figure S9. SAED patterns and TEM images of polycrystalline a) neat **SQ-H** (annealed at 150 ºC), b) BHJ with **PC$_{61}$BM** (annealed at 50 ºC), c) BHJ (annealed at 90 ºC), and d) BHJ (annealed at 130 ºC). e) Schematic of the two crystallites of **SQ-H** structure in BHJ thin films.

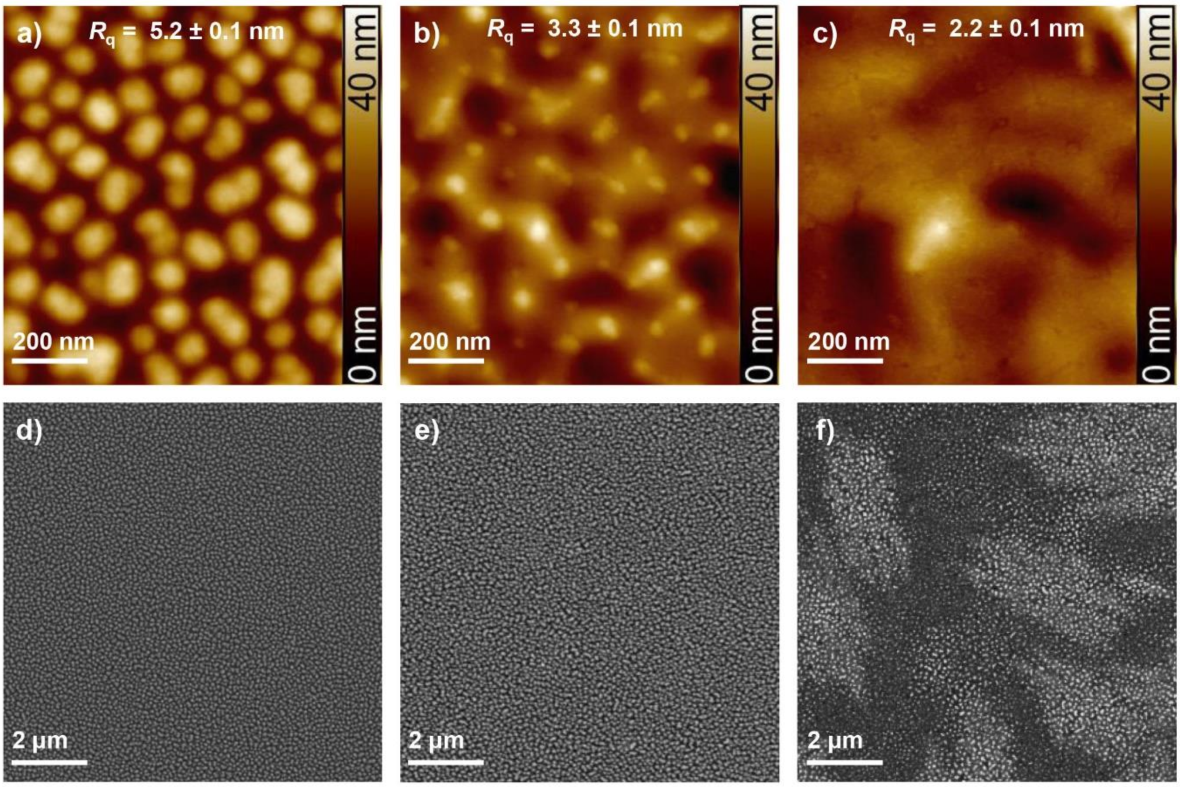

Figure S10. a-c) AFM height images and d-f) SEM images of the **SQ-H**:**$PC_{61}BM$** blend thin films on Si/SiO_2 substrates: a,d) as-cast and annealed b,e) at 90 °C, and c,f) at 130 °C. The root-mean-square roughness (Rq) values are denoted in the Figure.

Atomic force microscopy (AFM) and scanning electron microscope (SEM) analyses were carried out to find the optimum nano-morphology for **SQ-H**:**$PC_{61}BM$** active layers. With the increase of annealing temperature, AFM images show smaller-scale granular structures with reduced roughness, suggesting that phase separation between donor and acceptor occurs (Figure S10 a-c). The root mean square roughness values are 5.2 ± 0.1 nm, 3.3 ± 0.1 nm, and 2.2 ± 0.1 nm for as-cast, 90 ºC, and 130 ºC annealing conditions, respectively. Although higher temperature annealing conditions gave a smoother surface, which is generally beneficial for BHJ OPDs, significant phase separation occurs when the annealing temperature is increased to 130 ºC (Figure S10 d-e). Indeed, better EQE spectra were obtained from the OPD annealed at 90 °C compared to other annealing conditions (*vide supra*, Figure S11, Supporting Information).

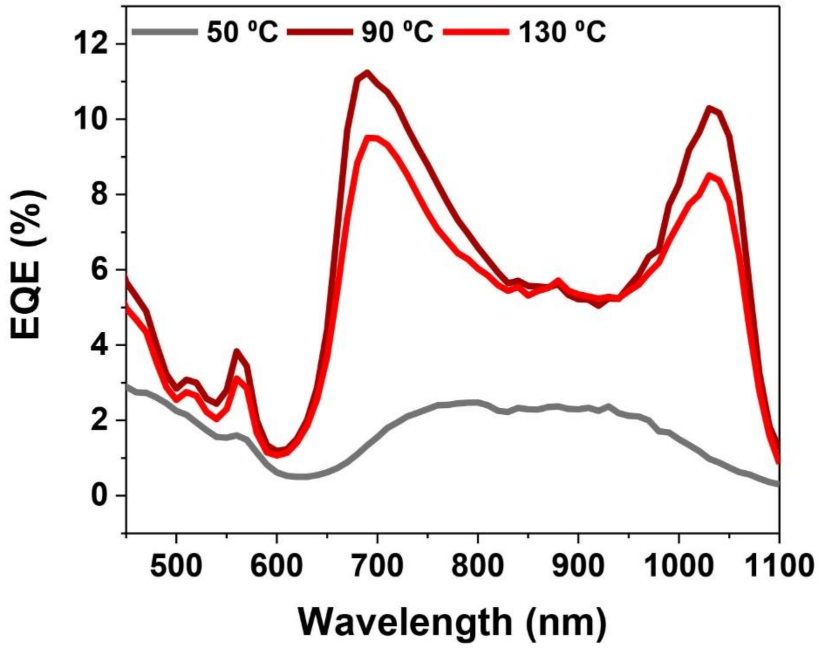

Figure S11. External quantum efficiency (EQE) of the **SQ-H**:**PC$_{61}$BM** OPDs depending on the annealing temperatures of the active layer.

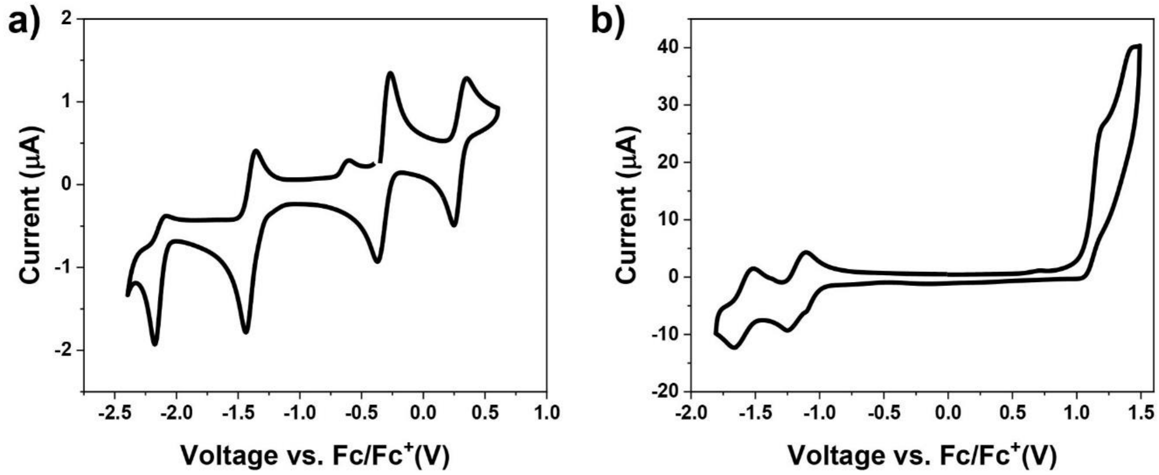

Figure S12. Cyclic voltammograms of a) SQ-H and b) $PC_{61}BM$ in CH_2Cl_2 using ferrocene/ferrocenium couple as an internal standard (scan rate : 100 mV s^{-1}). The oxidation potential ($E_{1/2}$(OX))/reduction potential ($E_{1/2}$(Red)) values of SQ-H and $PC_{61}BM$ are –0.32 V/–1.40 V and 1.18 V/–1.18 V, respectively. The HOMO and LUMO energy levels were estimated assuming a value of –5.1 eV for ferrocene.

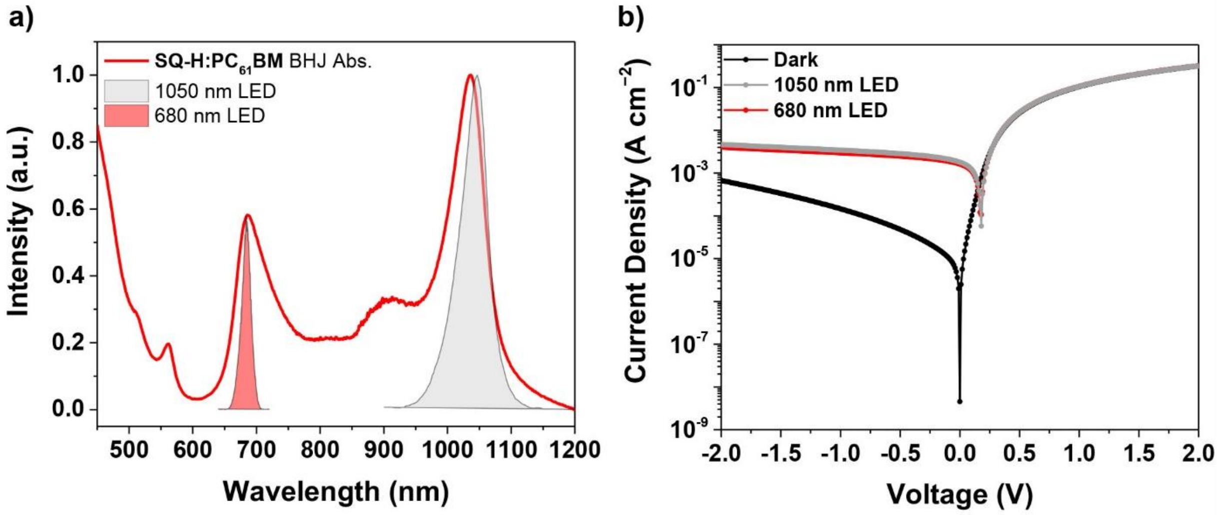

Figure S13. a) Absorption spectrum of annealed **SQ-H**:**PC$_{61}$BM** bulk-heterojunction thin-film and LED emission spectra with maximum emission peaks at 1050 nm (gray area) and 680 nm (red area). b) *J*–*V* curves of the OPD in the dark (black line) and under 1050 nm (gray line, ~15 mW cm^{-2}) and 680 nm (red line, ~15 mW cm^{-2}) illumination.

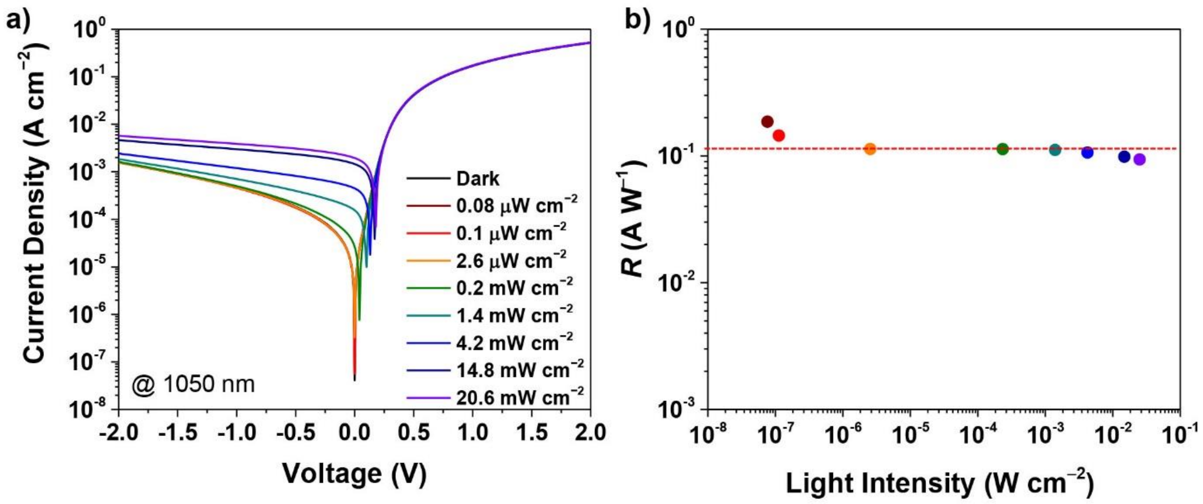

Figure S14. Photoresponse of the **SQ-H**:**$PC_{61}BM$** bulk-heterojunction OPD under the illumination of various NIR (1050 nm) light intensities. a) *J*-*V* characteristics of the **SQ-H**:**$PC_{61}BM$** bulk-heterojunction and b) corresponding responsivity (R).

Here, the responsivity (R) values are calculated by the following equation:

$$R = \frac{J_{\mathrm{ph}}}{P_{\mathrm{light}}}, \tag{S1}$$

where J_{ph} is the photocurrent density [A cm^{-2}], which is calculated from the *J*-*V* characteristics in Figure S14a, and P_{light} is the incident light intensity [W cm^{-2}]. The calculated R value in Figure S14b agrees well with the R for 1050 nm from EQE measurement (Figure S15b).

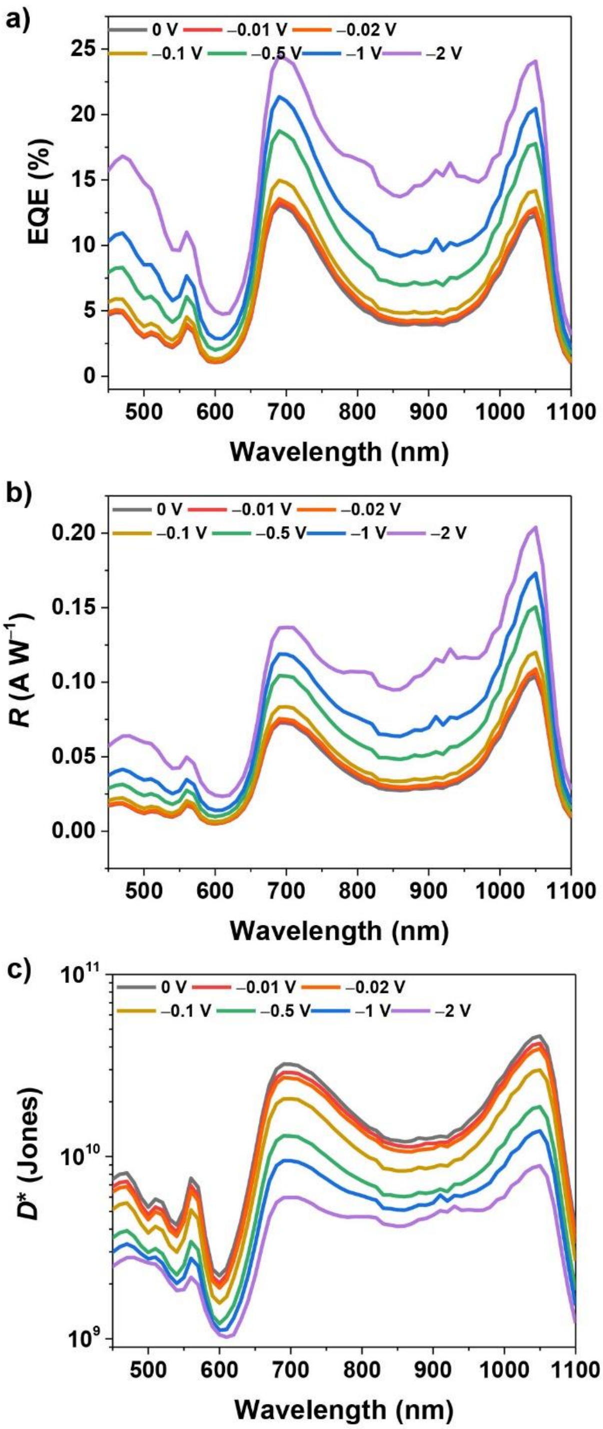

Figure S15. The spectral response of the **SQ-H**:$\mathbf{PC_{61}BM}$ bulk-heterojunction OPDs under reverse bias conditions (0 to −2 V). a) External quantum efficiency (EQE), b) responsivity (*R*), and c) detectivity (*D**) depending on the various reverse bias conditions.

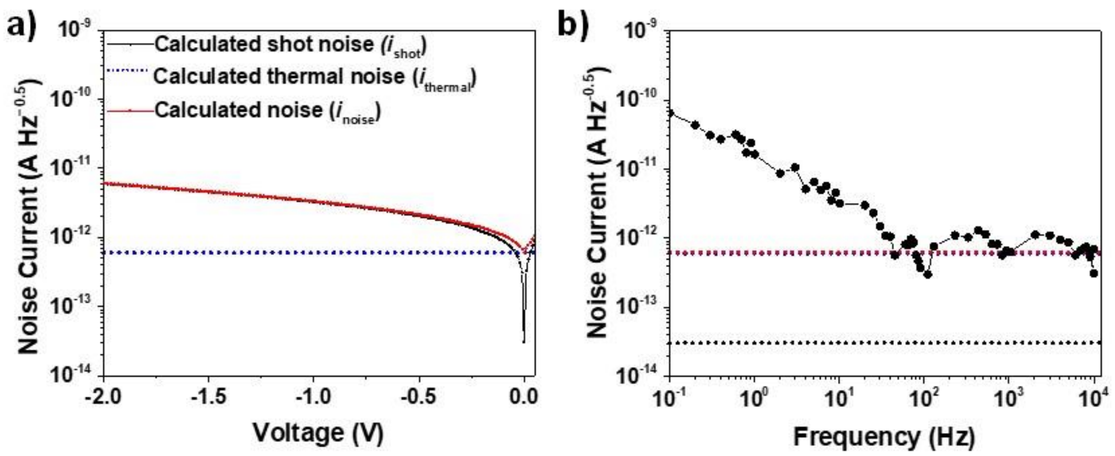

Figure S16. a) The calculated shot noise from the *J-V* characteristics of the **SQ-H**:**PC61BM** BHJ OPD. b) The experimental noise spectra of the OPD at 0 V. The black, blue, and red dotted lines are calculated shot noise, thermal noise, and noise, respectively, at 0 V.

The shot noise (i_{shot}) and thermal noise ($i_{thermal}$) were calculated using the following equations

$$i_{\text{shot}} = \sqrt{2qI_{\text{dark}}\Delta f}, \quad \text{(S2)}$$

$$i_{\text{thermal}} = \sqrt{\frac{4k_{\text{B}}T\Delta f}{R_{shunt}}}, \quad \text{(S3)}$$

where q is the elementary charge, I_{dark} is the dark current, Δf is the bandwidth, k_{B} is the Boltzmann constant, T is the temperature and R_{shunt} is the shunt resistance. I_{dark} and R_{shunt} from the *J-V* curve are 3 nA (@ 0V) and 44.7 kΩ, respectively. Assuming that shot noise and thermal noise are dominant factors of the noise current, the noise current can be calculated by the following equation:

$$i_{\text{noise}} \approx \sqrt{(i_{\text{shot}})^2 + (i_{\text{thermal}})^2} = \sqrt{(3 \times 10^{-14})^2 + (6 \times 10^{-13})^2} = 6 \times 10^{-13}\ \text{A Hz}^{-0.5}. \quad \text{(S4)}$$

Finally, the detectivity at 1050 nm under 0 V can be calculated by the following equation:

$$D^* = \frac{R\sqrt{A\Delta f}}{i_{\text{noise}}} = \frac{0.104 \times \sqrt{0.071}}{6 \times 10^{-13}} = 4 \times 10^{10}\ \text{Jones}. \quad \text{(S5)}$$

Note that several previously reported papers estimated detectivity as shot-noise limited detectivity (i.e., $i_{\text{noise}} \approx i_{\text{shot}}$) without noise spectral density investigation. This approach can

overestimate detectivity by orders of magnitude. For example, our optimized OPD showed shot-noise limited detectivity of 9×10^{11} Jones at 1050 nm under short-circuit condition.

To verify the calculated noise current and the detectivity values, the noise current spectral density was directly measured using a lock-in amplifier. For the frequency region between 30 Hz and 10 kHz, the noise current values were fluctuated between 1×10^{-12} A $Hz^{-0.5}$ (@ 30 Hz) and 3×10^{-13} A $Hz^{-0.5}$ (@ 110 Hz), see Figure S16b. Thus, the corresponding detectivity values are between 2×10^{10} Jones and 9×10^{10} Jones for 1050 nm, showing a similar magnitude to the calculated value (4×10^{10} Jones) from *J-V* curve.

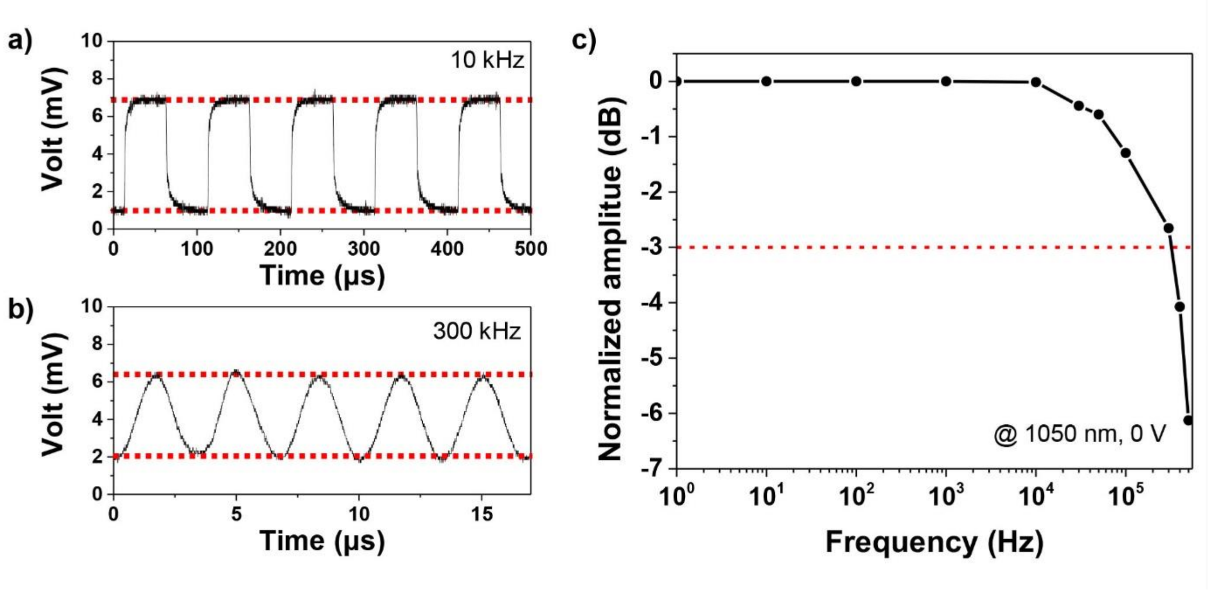

Figure S17. Phototransient response of the OPD under a) 10 kHz and b) 300 kHz modulated 1050 nm LED light at 0 V. c) Normalized photoresponse amplitude as a function of frequency.

The RC time constant-limited cutoff frequency (f_{RC}) can be calculated by the following equations[S6]

$$C = \varepsilon_0 \varepsilon_r \frac{A}{d}, \quad \text{(S6)}$$

$$f_{RC} = \frac{1}{2\pi RC}, \quad \text{(S7)}$$

where C is the capacitance, ε_0 is the vacuum permittivity, ε_r is dielectric constant (between 2 and 4 for typical organic semiconductors), A is the area of the active area, d is the thickness of the active layer, and R is the total series resistance of the device, which can be estimated to the sum of the series resistance of organic photodiode and the load resistance of the oscilloscope (50 Ω). Using $\varepsilon_0 = 8.854\times10^{-12}$ F m^{-1}, $\varepsilon_r = 3.5$, $A = 0.071$ cm^2 and $d = 85$ nm, the calculated C is range in 1.48 nF < C < 2.96 nF. The series resistance of our photodiode is estimated to be 40 Ω from the J-V curve, and thus R is calculated to be 90 Ω. Therefore, f_{RC} of our organic photodiode is range in 600 kHz < f_{RC} < 1.2 Mhz.

The transit time-limited cutoff frequency (f_{tr}) can be calculated by the following equations[S7]

$$\mu_{eff} = \frac{2\mu_e\mu_h}{\mu_e+\mu_h}, \quad \text{(S8)}$$

$$f_{\mathrm{tr}} = \frac{3.5}{2\pi\tau_{tr}} = \frac{3.5\mu_{eff}(V+V_{bi})}{2\pi d^2}, \quad \text{(S9)}$$

where μ_{eff} is the effective carrier mobility, μ_{e} is the electron mobility, μ_{h} is the hole mobility, τ_{tr} is the transit time of the charge carriers, V is the applied bias, V_{bi} is the built-in voltage, d is the thickness of the active layer. Using the SCLC mobilities (μ_{e} = 2×10^{-5} cm^2 V^{-1} s^{-1} and μ_{h} = 1×10^{-3} cm^2 V^{-1} s^{-1}), μ_{eff} is calculated to be 4×10^{-5} cm^2 V^{-1} s^{-1}. In other words, effective carrier mobility is governed by relatively low electron mobility of **$PC_{61}BM$**. Accordingly, f_{tr} is calculated to be 330 kHz, which is close to the experimental result (300 kHz).

Therefore, we assume that cutoff frequency of **SQ-H** OPD (300 kHz) is limited by f_{tr} (330 kHz) rather than f_{RC} (600 kHz < f_{RC} < 1.2 MHz).

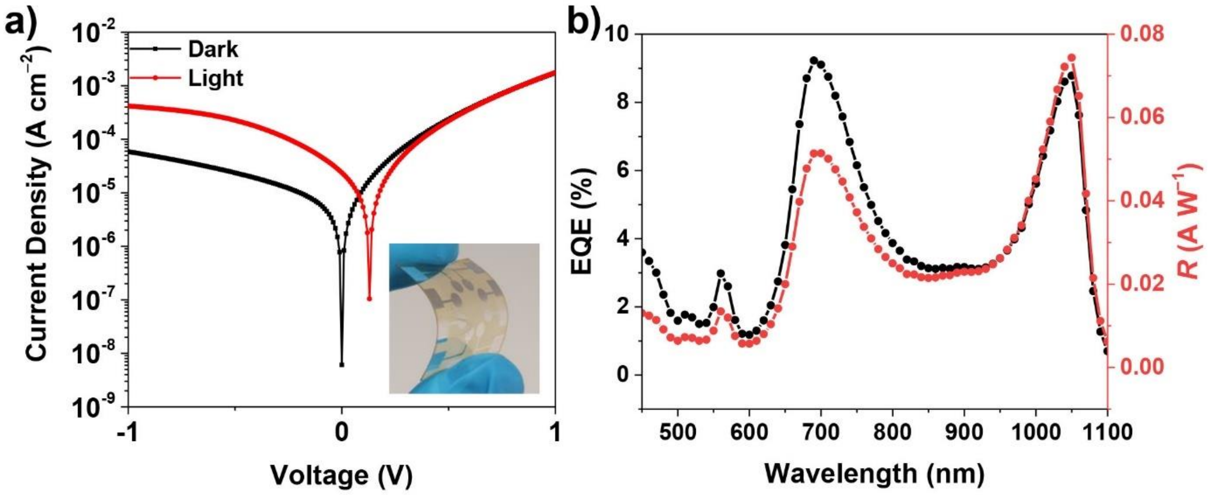

Figure S18. Flexible OPD characteristics**.** a) *J–V* curves of the OPD in the dark and under illumination with NIR light (~3 mW cm^{-2} @ 1050 nm). b) EQE and responsivity spectrum under 0 V.

Supporting Tables

Table S1. The references of the NIR OPDs devices in the Figure S1 with full-width at half-maximum (FWHM) values.

#	Active layer[a)]	$\lambda_{NIR,max}$ [nm][b)]	EQE [%][c)]	FWHM [nm][c)]	Reverse bias [V]	Active Area [mm^2]	Ref.
1	**SQ-H**:$PC_{61}BM$	1050	12.3	85	0	7.1	This work
2	**SQ-H**:$PC_{61}BM$	1050	20.5	91	−1	7.1	This work
3	**SQ-H**:$PC_{61}BM$	1050	24.1	>200	−2	7.1	This work
4	**DTBPh**:C_{60}	1045	1.2	>200	0	-	[S8]
5	**CNPh**:C_{60}	1130	1.6	>200	0	-	[S8]
6	**Psub**:C_{60}	1090	2.1	>200	0	-	[S8]
7	**Pfused**:C_{60}	1350	6.5	>250	0	-	[S8]
8	**Psub**: C_{60}	1150	3.6	>200	0	-	[S9]
9	**Pfused**: C_{60}	1400	10.5	>200	0	-	[S9]
10	**Heptamethine salt cation 1^+BF4$^-$**: C_{60}	1200	2.1	>500	0	5.7	[S10]
11	**Heptamethine salt cation 1^+TPFB$^-$**: C_{60}	1200	1.1	>500	0	5.7	[S10]
12	**Heptamethine salt cation 2^+BF4$^-$**: C_{60}	1350	1.4	>500	0	5.7	[S10]
13	**Heptamethine salt cation 2^+TPFB$^-$**: C_{60}	1350	0.8	>500	0	5.7	[S10]
14	**P3**:$PC_{71}BM$	1100	7	>500	0	9.0	[S11]
15	**P4**: $PC_{71}BM$	1200	6	>500	0	9.0	[S11]
16	**P5**: $PC_{71}BM$	1350	~1	>500	0	9.0	[S11]
17	**P1**(P4 of ref.[5]):$PC_{71}BM$	1100	17	>300	0	9.0	[S12]
18	**P1**(P4 of ref. [5]): $PC_{71}BM$ +Camphoric anhydride	1100	26	>300	0	9.0	[S12]
19	**P2**:$PC_{71}BM$	1100	3.8	>300	0	9.0	[S12]
20	**P2**:$PC_{71}BM$ +Camphoric anhydride	1100	7.8	>300	0	9.0	[S12]
21	**PBBTPD**:Tri-$PC_{61}BM$	1200	~16	>500	−0.5	4.5	[S13]
22	DPP-DTT:**SWIR dy**e	1100	~0.45	>300	−0.05	10&150	[S14]
23	**PTTQ(BO)**:$PC_{71}BM$	1145	~18	>400	−2	3	[S15]
24	**PTTQ(BO)**:$PC_{71}BM$	1145	~10	>400	0	3	[S15]
25	**PTTQ(HD)**:$PC_{71}BM$	1117	~6	>400	−2	3	[S15]

26	**PTTQ(HD)**:$PC_{71}BM$	1117	~4	>400	0	3	[S15]
27	**PTTTQ(OD)**:$PC_{71}BM$	1053	~8	>400	−2	3	[S15]
28	**PTTTQ(OD)**:$PC_{71}BM$	1053	~6	>400	0	3	[S15]
29	**PBiTTQ(HD)**:$PC_{71}BM$	1015	~12	>300	−2	3	[S15]
30	**PBiTTQ(HD)**:$PC_{71}BM$	1035	~5	>300	0	3	[S15]
31	**PBiTTQ(OD)**:$PC_{71}BM$	1018	~7	>300	−2	3	[S15]
32	**PBiTTQ(OD)**:$PC_{71}BM$	1018	~6	>300	0	3	[S15]

[a)]NIR absorbing components are highlighted in bold; [b,c,d)] Several values are estimated from reported EQE or responsivity spectra.

Table S2. Parameters for modeling the absorption spectra of **SQ-H** in Figure 3.

**Parameter**	**Definition**	**Values**	
		**Monomer**	**Dimer**
η_Z	Energy difference between neutral and zwitterionic state	1560 cm^{-1}	1560 cm^{-1}
t_Z	Electronic coupling between neutral and zwitterionic state	7430 cm^{-1}	7430 cm^{-1}
ω_0	Vibrational frequency	1300 cm^{-1}	1300 cm^{-1}
vib_{max}	Maximal number of total vibrational quanta for each chromophore	6	6
λ_Z^2	Huang-Rhys factor for zwitterionic state	0.86	0.86
η_{CT}	Energy difference between neutral and intermolecular charge transfer (CT) state	–	7800 cm^{-1}
t_{CT}	Electronic coupling between neutral and intermolecular CT state	–	4300 cm^{-1}
λ_{CT}^2	Huang-Rhys factor for intermolecular CT state	–	0.36
ε_r	Screening factor for Coulomb interaction	–	2.9
ΔE	Overall spectral shift arising from polarizable surroundings	–	–850 cm^{-1}
σ	Full width at 1/e height of absorbance	350 cm^{-1}	350 cm^{-1}

Table S3. Summary of optical properties of **SQ-H** in CH_2Cl_2 solution and their spin-coated neat and BHJ (**SQ-H**:**PC$_{61}$BM**=1:1.5 weight ratio) thin films on quartz substrates.

	$\lambda_{Abs.peak1}$ [a)] [nm]	FWHM$_{peak1}$ [b)] [nm/cm^{-1}]	$\lambda_{Abs.peak2}$ [a)] [nm]	FWHM$_{peak2}$ [b)] [nm/cm^{-1}]	λ_{PL} [c)] [nm]	FWHM$_{PL}$ [d)] [nm/cm^{-1}]	$\Delta\tilde{\nu}_{Stokes}$ [e)] [nm/cm^{-1}]
**Solution**	870	44/584	–	–	–	–	–
**As-cast thin-film**	799	–	909	237/3278	–	–	–
**Annealed thin-film**	688	65/1335	1040	59/555	1050	44/400	10/90
**Annealed BHJ thin-film**	688	73/1496	1040	71/676	–	–	–

[a)]Absorption peaks in absorption spectrum and [b)]corresponding full width at half maximum; [c)]Maximum emission peak in photoluminescence spectrum and [d)]corresponding full width at half maximum; [e)]Stokes shift.

Table S4. The charge transfer integral values for hole (t_h) and electron (t_e) carriers of the nearest-neighbor dimers

**Dimer**[a)]	t_h [meV] [b)]	t_e [meV] [c)]
D1	253	−93
D2	8	0
D3	−20	0

[a)] The nearest-neighbor dimers in **SQ-H** single crystal structure (Figure S5c); [b,c)] Charge transfer integral values were calculated using PW91 functional and TZP basis sets implemented Amsterdam Density Functional package.

Table S5. The characteristics of the **SQ-H**:**PC$_{61}$BM**-based bulk-heterojunction OPDs with various reverse bias conditions.

Reverse Bias [V]	*EQE*@690 nm [%]	*R*@690 nm [A W^{-1}]	*D**@690 nm [Jones]	*EQE*@1050 nm [%]	*R*@1050 nm [A W^{-1}]	*D**@1050 nm [Jones]
0	13.07	0.073	3.22×10^{10}	12.26	0.104	4.59×10^{10}
0.01	13.24	0.074	2.90×10^{10}	12.55	0.106	4.18×10^{10}
0.02	13.57	0.075	2.71×10^{10}	12.85	0.109	3.91×10^{10}
0.1	14.96	0.083	2.07×10^{10}	14.17	0.120	2.99×10^{10}
0.5	18.75	0.104	1.30×10^{10}	17.78	0.151	1.88×10^{10}
1	21.36	0.119	9.50×10^{9}	20.45	0.173	1.38×10^{10}
2	24.51	0.136	5.96×10^{9}	24.08	0.204	8.91×10^{9}

Table S6. Crystal data and structure refinement for **SQ-H**.

**Compounds**	**SQ-H**
CCDC number	2057832
Chemical formula	$C_{51}H_{64}N_4O$
M / g mol^{-1}	749.06
T / K	300
Crystal description	plate
Crystal color	green
Crystal system	Monoclinic
Space group	P 2/c
a / Å	6.6946(3)
b / Å	9.6069(5)
c / Å	34.7009(16)
α / °	90
β / °	90.215(4)
γ / °	90
V / Å^3	2231.75(18)
Z	2
ρ_{cal} / g cm^{-3}	1.115
μ / mm^{-1}	0.504
F(000)	812
Measurement range of θ / °	2.547-75.254
Completeness / %	99.9
Number of independent reflections	4569
Parameters/restraints	256/0
Goodness of fit for F^2	1.039
$R[I > 2\sigma(I)]$	R_1 = 0.0522; $_wR_2$ = 0.1382
R(all data)	R_1 = 0.0753; $_wR_2$ = 0.1555

Supporting References

[S1] U. Mayerhöffer, B. Fimmel, F. Würthner, *Angew. Chem. Int. Ed.* **2012**, *51*, 164; c) U. Mayerhöffer, F. Würthner, *Angew. Chem. Int. Ed.* **2012**, *51*, 5615.

[S2] M. J. Frisch, G. W. Trucks, H. B. Schlegel, G. E. Scuseria, M. A. Robb, J. R. Cheeseman, G. Scalmani, V. Barone, G. A. Petersson, H. Nakatsuji, X. Li, M. Caricato, A. V. Marenich, J. Bloino, B. G. Janesko, R. Gomperts, B. Mennucci, H. P. Hratchian, J. V. Ortiz, A. F. Izmaylov, J. L. Sonnenberg, Williams, F. Ding, F. Lipparini, F. Egidi, J. Goings, B. Peng, A. Petrone, T. Henderson, D. Ranasinghe, V. G. Zakrzewski, J. Gao, N. Rega, G. Zheng, W. Liang, M. Hada, M. Ehara, K. Toyota, R. Fukuda, J. Hasegawa, M. Ishida, T. Nakajima, Y. Honda, O. Kitao, H. Nakai, T. Vreven, K. Throssell, J. A. Montgomery Jr., J. E. Peralta, F. Ogliaro, M. J. Bearpark, J. J. Heyd, E. N. Brothers, K. N. Kudin, V. N. Staroverov, T. A. Keith, R. Kobayashi, J. Normand, K. Raghavachari, A. P. Rendell, J. C. Burant, S. S. Iyengar, J. Tomasi, M. Cossi, J. M. Millam, M. Klene, C. Adamo, R. Cammi, J. W. Ochterski, R. L. Martin, K. Morokuma, O. Farkas, J. B. Foresman, D. J. Fox, *Gaussian 16, Revision A.03,* Gaussian, Inc., Wallingford, CT 2016.

[S3] N. J. Hestand, C. Zheng, A. R. Penmetcha, B. Cona, J. A. Cody, F. C. Spano, C. J. Collison, *J. Phys. Chem. C* **2015**, *119*, 18964.

[S4] a) G. te Velde, F. M. Bickelhaupt, E. J. Baerends, C. Fonseca Guerra, S. J. A. van Gisbergen, J. G. Snijders, T. Ziegler, *J. Comput. Chem.* **2001**, *22*, 931; b) ADF2013, SCM, Theoretical Chemistry, Vrije Universiteit, Amsterdam, The Netherlands, http://www.scm.com

[S5] G. Sheldrick, *Acta Crystallogr. A* **2008**, *64,* 112.

[S6] N. Gasparini, A. Gregori, M. Salvador, M. Biele, A. Wadsworth, S. Tedde, D. Baran, I. McCulloch, C. J. Brabec, *Adv. Mater. Technol.* **2018**, *3,* 1800104.

[S7] J. Huang, J. Lee, J. Vollbrecht, V. V. Brus, A. L. Dixon, D. X. Cao, Z. Zhu, Z. Du, H. Wang, K. Cho, G. C. Bazan, T.-Q. Nguyen, *Adv. Mater.* **2020**, *32*, 1906027

[S8] J. D. Zimmerman, V. V. Diev, K. Hanson, R. R. Lunt, E. K. Yu, M. E. Thompson, S. R. Forrest, *Adv. Mater.* **2010**, *22*, 2780.

[S9] J. D. Zimmerman, E. K. Yu, V. V. Diev, K. Hanson, M. E. Thompson, S. R. Forrest, *Org. Electron.* **2011**, *12*, 869.

[S10] M. Young, J. Suddard-Bangsund, T. J. Patrick, N. Pajares, C. J. Traverse, M. C. Barr, S. Y. Lunt, R. R. Lunt, *Adv. Optical Mater.* **2016**, *4*, 1028.

[S11] A. E. London, L. Huang, B. A. Zhang, M. B. Oviedo, J. Tropp, W. Yao, Z. Wu, B. M. Wong, T. N. Ng, J. D. Azoulay, *Polym. Chem.* **2017**, *8*, 2922.

[S12] Z. Wu, Y. Zhai, W. Yao, N. Eedugurala, S. Zhang, L. Huang, X. Gu, J. D. Azoulay, T. N. Ng, *Adv. Funct. Mater.* **2018**, *28*, 1805738.

[S13] L. Zheng, T. Zhu, W. Xu, L. Liu, J. Zheng, X. Gong, F. Wudl, *J. Mater. Chem. C* **2018**, *6*, 3634.

[S14] N. Li, Z. J. Lan, Y. S. Lau, J. J. Xie, D. H. Zhao, F. R. Zhu, *Adv. Sci.* **2020**, *7*, 2000444.

[S15] F. Verstraeten, S. Gielen, P. Verstappen, J. Raymakers, H. Penxten, L. Lutsen, K. Vandewal, W. Maes, *J. Mater. Chem. C* **2020**, *8*, 10098.
